# Plasticity-Driven Regeneration of Circumvallate Papilla After Lgr5+ Stem Cells Loss

**DOI:** 10.7150/ijbs.130382

**Published:** 2026-05-11

**Authors:** Senthil Kumar Baskaran, Anish Ashok Adpaikar, Suyeon Lee, Junsu Kim, Yeo Song Lee, JooHyun Jee, Hidenori Akutsu, Thantrira Porntaveetus, Ki Woo Kim, Eun-Jung Kim, Jong-Min Lee, Han-Sung Jung

**Affiliations:** 1 Division in Anatomy and Developmental Biology, Department of Oral Biology, Taste Research Center, Oral Science Research Center, BK21 FOUR Project, Yonsei University College of Dentistry, Seoul, South Korea.; 2 Center for Regenerative Medicine, National Center for Child Health and Development, 2-10-1 Okura, Setagaya, Tokyo 157-8535, Japan.; 3 Center of Excellence in Precision Medicine and Digital Health, Department of Physiology, Faculty of Dentistry, Chulalongkorn University, Bangkok 10330, Thailand.; 4 Division of Physiology, Department of Oral Biology, BK21 FOUR Project, Yonsei University College of Dentistry, Seoul, 03722, Republic of Korea.

## Abstract

Taste receptor cells located within the papillae of the oral cavity have a defined lifespan of 8-12 days. They are continuously replenished by a pool of stem/progenitor cells residing outside the taste buds. Previous studies have identified Lgr5 positive stem/progenitor cells localized within the basal trench of the circumvallate papilla (CVP) as the source of all taste and non-taste epithelial cells. However, it remains unclear whether an alternative stem cell population within the CVP can regenerate the epithelium in the absence of Lgr5-expressing cells. Using Lgr5^DTR^ mice in which Lgr5-expressing cells were selectively ablated via diphtheria toxin administration, we observed a substantial loss of these cells in the CVP, leading to rapid degeneration of taste buds, K8-expressing taste receptor cells, basal cells, and the basement membrane within 24 h post-injection. Notably, while the basal cells and basement membrane were fully regenerated at 72 h post-injection, taste buds reappeared at 2 weeks. Previous studies have demonstrated that SOX10-Cre-labeled cells originating from Von Ebner's gland (VEG) contribute to the CVP epithelium and are detected within the taste bud. However, the cellular dynamics and mechanistic role of these SOX10-Cre-labeled cells in the maintenance of taste buds remain unclear. Lineage tracing results revealed that K8/K14-expressing cells from the ducts of VEG contribute to the regeneration of taste buds, indicating that these duct-associated epithelial cells contribute to regeneration, potentially through injury-induced cellular plasticity in the absence of a resident Lgr5-expressing stem cell population.

## Introduction

In recent years, numerous reports have highlighted the concept of cellular plasticity and the involvement of non-traditional stem cell populations in tissue regeneration. This process of injury-induced cellular plasticity allows both adult stem cells and differentiated cells to acquire new characteristics and regenerate organs 1-4. Specifically, Leucine-rich repeat-containing G-protein coupled receptor 5 positive (Lgr5+) cells are recognized as stem cells in various epithelial organs, playing a key role in maintaining homeostasis. However, previous studies conducted on the intestine, colon, and hair follicles have shown that Lgr5 negative cells can revert to a stem cell state through dedifferentiation, and they can persist until regeneration occurs 5-7.

Taste buds, located within the taste papillae on the surface of the tongue, consist of specialized sensory epithelial cells crucial for gustatory perception. In the posterior part of the tongue, taste receptor cells experience continuous turnover, which is sustained by Lgr5+ cells found in the circumvallate papilla (CVP). These progenitor cells generate new taste receptor cells and non-taste lingual epithelial cells under normal conditions in a nerve-dependent manner. Notably, when the innervating nerve is transected, it leads to the degeneration of the taste bud 8, 9. However, epithelial dedifferentiation of committed taste receptor cells has been observed during the regeneration process following nerve transection in the CVP 10.

Previous study has demonstrated that both the CVP taste buds and the underlying Von Ebner's gland (VEG) originate from a common Lgr5+ stem cell population. Lineage tracing of Lgr5+ cells revealed that, 1 month after tamoxifen induction, their progeny populates taste receptor cells, non-taste lingual epithelial cells, and VEG ductal cells, indicating a shared developmental origin and multipotent contribution of Lgr5+ progenitors to the posterior tongue epithelium 11. Lineage-tracing using Sox10-Cre mouse models has demonstrated that epithelial progenitors from the VEG form Keratin 8 (K8)-positive taste receptor cells within the CVP epithelium 12, 13. These findings indicate that perigemmal non-taste bud epithelial populations may act as an alternative source of taste receptor cells under certain conditions.

While Sox2 is a well-established marker of taste progenitor cells and Sox9 marks stem cell populations in glandular tissues, Sox10 represents a distinct lineage-associated marker primarily linked to neural crest-derived and ductal epithelial populations.

Unlike Sox2+ progenitors that actively maintain taste bud homeostasis, Sox10+ cells are predominantly localized in the VEG duct and have been implicated in glandular epithelial lineages, suggesting a potential role as a non-canonical or reserve progenitor population 12-14. Given their anatomical localization, lineage origin, and reported contribution to taste bud cells, Sox10+ ductal cells represent a compelling candidate population that may be activated upon injury and contribute to epithelial regeneration. However, it remains unclear whether Sox10+ cells function as an independent progenitor population, overlap with Sox2+ or Sox9+ cells, or occupy a distinct position within the regenerative hierarchy.

However, we still do not fully understand how Sox10-derived cells contribute to the maintenance of taste buds during normal conditions, nor how they are activated in response to injury-induced loss of resident stem cells. In particular, the relationship between this VEG-derived lineage and the well-characterized Lgr5+ stem cell population within the CVP remains unclear. We still need to determine whether Sox10-derived cells can compensate for taste bud regeneration after the targeted ablation of Lgr5+ cells. Clarifying the cellular origin, activation signals, and lineage plasticity of these Sox10-labeled cells is crucial for understanding the mechanisms of epithelial regeneration and the maintenance of taste buds in the posterior tongue.

Lgr5+ stem cells play a crucial role in maintaining taste buds and the homeostasis of the CVP. Consequently, substantial loss of this population serves as an experimental model to assess the compensatory regenerative mechanisms that may come into play during severe epithelial damage. This is similar to what occurs in the intestine, where non-Lgr5+ cells can transiently acquire stem-like properties 4, 7.

Lgr5+ cells typically reside in the crypt region of the intestine or in hair bulges within the hair follicle 15, 16. In contrast, CVP possesses Lgr5+ cells in the crypt and perigemmal regions of the taste bud. Therefore, the Lgr5+ cells ablation in the CVP is severe, leading to morphological disruption in half of the organ. These Lgr5+ cells are the only known stem cell population that supports the development, maintenance, and regeneration of taste buds. Consequently, substantial loss of this cell lineage provides a model to investigate whether compensatory regenerative mechanisms can be activated in response to severe epithelial injury. This scenario is similar to what occurs in the intestine, where non-Lgr5+ progenitors can temporarily acquire stem-like properties. Although such complete depletion is rare in physiological conditions, this model reveals latent regenerative plasticity within surrounding tissues, providing insights into the mechanisms of cross-tissue compensation during epithelial repair. Given the expression pattern of Lgr5+ cells in the CVP, we aimed to elucidate the origin and mechanism of CVP regeneration following Lgr5+ cell ablation. In this study, we demonstrated that two distinct organs, the CVP and VEG, develop from the exact origin and temporally share stem cells that respond to CVP regeneration after injury. The fundamental importance of these dynamic epithelial interactions in acute injury states would enable us to harness an unlimited capacity for unknown regenerative processes.

## Materials and Methods

### Animal

All animal procedures were conducted in compliance with institutional and national guidelines for the care and use of laboratory animals. Experimental protocols were reviewed and approved by the Yonsei University Health System Institutional Animal Care and Use Committee (YUHS-IACUC; approval no. 2023-0053), and were performed in accordance with the guidelines established by the National Research Council (USA). Mice were maintained under controlled environmental conditions, including a constant temperature of 22 °C, relative humidity of 55%, and a 12-hour light/12-hour dark cycle (lights on from 05:00 to 17:00). Food and water were provided *ad libitum*. All experimental procedures were carried out under deep anesthesia to minimize animal distress. Adult mice were obtained from Koatech (Pyeongtaek, Korea) and acclimated to the housing conditions prior to experimentation. Mouse models used in this study for Lgr5+ cell ablation were Lgr5^DTR-EGFP/+ 7^. For tracing the fate of stem cells, *Lgr5*^EGFP-IRES-CreERT2 15^, progenitor cells *K14*^CreERT/+ 17^, and differentiated taste receptor cells *K8*^CreERT2/+^ 18 mice were bred with *R26^flox-STOP-tdTomato^* 19 to generate *Lgr5*^EGFP-CreERT2/+;^ *R26R*^tdT/+^, *K14*^CreERT/+;^ *R26R*^tdT/+^ and *K8*^CreERT2/+^; *R26R*^tdT/+^. At least 6 adult mice (>6 w of age) were used for each experiment.

### Lgr5 ablation

The Diphtheria toxin (DT) (D0564, Sigma) was prepared as described previously 20. The *Lgr5*^DTR^ mice were injected with 50 μg/kg DT via intraperitoneal injection. The CVP from the *Lgr5*^DTR^ mice was harvested at different time points after a single DT administration.

### Lineage tracing

All transgenic mouse strains utilized in this study have been previously characterized and were maintained on a C57BL/6 genetic background. To induce Cre-dependent recombination, tamoxifen (T5648, Sigma-Aldrich, St. Louis, MO) was administered via intraperitoneal injection at 100 mg/kg body weight for two consecutive days. For diphtheria toxin-mediated ablation experiments, mice received a single dose of DT and were euthanized at the designated time points.

### BrdU labeling

5-bromo-2-deoxyuridine (BrdU, B5002, Sigma, MO, USA) was injected for five consecutive days after DT injection. The labeled mice were sacrificed at 3 months from the last BrdU injection to identify label-retaining cells (LRCs) in the VEG duct. For the identification of reversible activation of the LRCs in the VEG duct, the mice were injected with DT and BrdU as described above. 2 w later, the mice were again injected with DT and 5-iodo-2′-deoxyuridine (IdU, I7125, Sigma, MO, USA) was injected into the mice to identify the reversible activation of the LRCs in the duct. At least 6 mice were used for each experiment.

### Histology and immunofluorescence

Tissue samples were fixed in 4% paraformaldehyde and processed using standard histological procedures. Paraffin-embedded tissues were sectioned at a thickness of 5 µm for hematoxylin and eosin (H&E) staining as well as immunohistochemical analysis. For antigen retrieval, sections were subjected to heat-induced epitope retrieval in citrate buffer (pH 6.0). Non-specific binding was minimized by blocking with either 1% goat serum or 5% bovine serum albumin (BSA) in phosphate-buffered saline (PBS). Sections were then incubated with primary antibodies against Gα Gustducin (sc-395, Santa Cruz Biotechnology, Inc., USA, 1:100), SNAP25 (sc-20038, Santa Cruz Biotechnology, Inc., USA, 1:100), BrdU (ab6326, Abcam, UK, 1:100), c-Kit (ab231780, Abcam, UK; 1:100), PCNA (ab18197, Abcam, UK; 1:200), RFP (600-401-379, Rockland, USA, 1:200), K14 (ab7800, Abcam, UK, 1:200), K8 (TROMA-I, DSHB, IA, USA, 1:200), Trpm5 (Guinea pig polyclonal anti-Trpm5 antibody was generated against amino acids residues 1,089-1,158 of Trpm5), MMP9 (AB19016, Merck Millipore, USA, 1:200), Beta-catenin (sc-7963 Santa Cruz Biotechnology, Inc., USA, 1:200), Hes1 (ab108937, Abcam, UK, 1:200), p70 S6 Kinase Antibody (9202, Cell Signaling Technology, USA, 1:200), p70 S6 Kinase Antibody T369(AP0564, ABclonal, USA, 1:200), p70 S6 Kinase Antibody T421/S424 (AP0502, ABclonal, USA, 1:200), Lef1 (2230S, Cell Signaling Technology, USA, 1:200), Rock1 (ab45171, Abcam, UK, 1:200), Rock2 (610623, BD Bioscience, USA, 1:200) and HDAC2 (D6S5P) (57156, Cell Signaling Technology, USA, 1:200) at 4 °C overnight. Following primary antibody incubation, sections were exposed to appropriate secondary antibodies (1:200; Invitrogen, USA). Nuclear counterstaining was performed using either TO-PRO™-3 (T3605; Invitrogen, USA; 1:1000) or DAPI (D1306; Invitrogen, USA; 30 nM). Fluorescence signals were visualized and captured using a confocal laser scanning microscope (DMi8; Leica Microsystems, Germany).

### Transmission electron microscope (TEM)

Samples were fixed for 12 h in a mixture of 2% glutaraldehyde and 2% paraformaldehyde prepared in 0.1 M phosphate buffer (pH 7.4), followed by rinsing in the same buffer. Post-fixation was carried out using 1% osmium tetroxide (OsO₄) in 0.1 M phosphate buffer for 2 h. After fixation, tissues were dehydrated through a graded ethanol series (50-100%) and subsequently treated with propylene oxide prior to resin infiltration. Specimens were then embedded in epoxy resin (Poly/Bed 812; Polysciences) and polymerized at 65 ºC for 12 h in a drying oven. Semi-thin sections (~200 nm) were obtained using an ultramicrotome (UC7; Leica Microsystems, Vienna, Austria) equipped with a diamond knife and stained with toluidine blue for light microscopic examination. Regions of interest were further sectioned into ultrathin sections (~80 nm), mounted on copper grids, and contrasted with uranyl acetate followed by lead citrate. Ultrastructural images were acquired using a transmission electron microscope (HT7800; Hitachi, Tokyo, Japan) operated at an accelerating voltage of 80 kV.

### Bulk RNA-seq analysis

Strand-specific RNA-seq libraries were constructed using the TruSeq Stranded mRNA Sample Preparation Kit (Illumina, CA, USA) and sequenced with a 151-bp paired-end configuration.

Poly(A)+ RNA was isolated from 1 µg of total RNA using oligo (dT) magnetic beads, followed by fragmentation. First-strand cDNA synthesis was performed using random hexamer primers, and second-strand synthesis generated double-stranded cDNA. Following end repair, A-tailing, and adaptor ligation, libraries were amplified by PCR. Library quality was assessed using an Agilent 2100 Bioanalyzer (Agilent, CA, USA), and concentrations were determined with a KAPA Library Quantification Kit (Kapa Biosystems, MA, USA) according to the manufacturer's instructions. Following cluster amplification of denatured templates, paired end sequencing was conducted (2×151bp) using an Illumina NovaSeq6000 (Illumina, CA, USA).

Adapter sequences as well as ends of reads less than a Phred quality score of 20 were trimmed, and reads shorter than 50 bp were removed using cutadapt v.2.8 21.

Quality-filtered reads were aligned to the appropriate reference genome using STAR (v2.7.1a) 22 with parameters consistent with ENCODE guidelines. To facilitate transcript-level quantification, the “-quantMode TranscriptomeSAM” option was applied during alignment.

Gene expression estimated using the featureCounts function of the Subread package v2.0.8 23. Normalization and transformation of gene expression and differentially expressed gene (DEG) analysis were performed using DESeq2 v1.46.0 24. The threshold of significance for DEG analysis was set as the adjusted p-Value < 0.05 (Benjamini-Hochberg method) and | Log_2_ (Fold change) | < Log_2_ 1.5. Gene ontology (GO) terms enriched on the significant DEGs were accessed using cluster Profiler v4.14.3 25. Volcano plots, bar plots, dot plots and heatmaps were generated using ggplot2 v3.5.1 and ComplexHeatmap v2.22.0 26. The statistical analysis and visualization of Bulk RNA-seq data were performed by NGeneS Inc. (Ansan-si, Republic of Korea).

### Single-cell RNA sequencing data processing and analysis

The single cell RNA sequencing dataset of the epithelium of Sox10-Cre/tdT+ mouse CVP/VEG complex 13 was obtained from NCBI GEO repository (accession number GSE273518). Raw single cell RNA sequencing data, including the barcode, feature, and matrix files, were retrieved from the GEO repository. The data were imported into R and processed using the Seurat package v5.2.1 27, starting with the creation of a Seurat object for downstream analysis. To ensure data quality, cells were filtered based on the following criteria: number of detected genes per cell (nFeature_RNA) > 500, total RNA counts per cell (nCount_RNA) > 500, and mitochondrial gene content < 10%. After filtering, 2,215 high-quality cells were retained from the initial 16,548 cells. Gene expression values were log-transformed and normalized using the LogNormalize method in Seurat, ensuring that the expression distributions were comparable across cells. Principal component analysis (PCA) was conducted using the RunPCA function, and dimensionality reduction was performed via Uniform Manifold Approximation and Projection (UMAP) using RunUMAP function (dims = 1:15). Cell clustering was achieved through FindNeighbours function to determine intercellular distances, followed by FindClusters with a resolution of 0.5. Cell populations were subsequently annotated based on the annotation of the cell type (ACT) database 28. For trajectory inference, lineage reconstruction was performed using SlingShot, a R package for cell lineage and pseudotime inference for single-cell transcriptomics 29. The slingshot function was applied to the UMAP embeddings with cluster 16 specified as the root cluster. The smoothing parameter omega was enabled and scaled by a factor of 4 to refine the lineage curve. The statistical analysis and visualization of scRNA-seq data were performed by NGeneS Inc. (Ansan-si, Republic of Korea).

### *In situ* hybridization

All samples were fixed in 4% paraformaldehyde in PBS, and 5 μm-thick paraffin sections were prepared under RNase-free conditions for *in situ* hybridization. *In situ* hybridization for Lgr5 was performed using RNAscope 2.5 Assay (Advanced Cell Diagnostics, ACD, United States) according to the manufacturer's protocols 30. RNAscope probes targeting Lgr5 were designed and validated by Advanced Cell Diagnostics (ACD). Paraffin-embedded sections were deparaffinized and subjected to heat-mediated target retrieval, followed by protease treatment. Sections were subsequently hybridized with Lgr5-specific oligonucleotide probes according to the manufacturer's instructions. Color development was performed with Fast Red substrate. Intracellular red punctate dots are considered a positive signal.

### Organoid culture

Tongues were harvested from adult mice following euthanasia and injected with ~0.5 mL of Dispase II (4942078001, Roche, USA) diluted in PBS, followed by incubation at 37 ºC for 25 min. The epithelial layer was then carefully separated from the underlying mesenchyme.

Epithelia from the circumvallate and foliate papillae were isolated and further dissociated using TrypLE™ (12604013, Gibco, Denmark) at 37 ºC for 30 min. Cells were pelleted by centrifugation (800 rpm, 20 min) and resuspended in growth factor-reduced Matrigel (356234, Corning, USA). Cell suspensions were plated as 50 µL domes in 24-well culture plates and allowed to solidify at 37 ºC for at least 10 min. Organoids were maintained in taste culture medium based on DMEM/F12 (11320033, Gibco, USA) supplemented with N2 (17502048, Gibco, MA, USA), B27 (17504044, Gibco, MA, USA), R-spondin-1 (120-38, Peprotech, NJ, USA, 200 ng/mL,), Noggin (120-10C, NJ, USA,100 ng/mL), Jagged-1 (188-204, Anaspec, CA, USA, 1 μM,), Y27632 (1254, Tocris, Korea, 1 μM), N-acetylcysteine (A7250, Sigma, MO, USA 1 mM), epidermal growth factor (AF-100-15, Peprotech, NJ, USA, 50 ng/mL) was added to the plate. Growth media was changed every 3 days. Addition of DT to the media was performed as previously described 31.

### RT-qPCR

Total RNA was isolated using TRIzol® reagent (#15596-026, Thermo Fisher Scientific, USA). Complementary DNA (cDNA) was synthesized using Maxime RT PreMix (#25081, iNtRON, Korea) according to the manufacturer's instructions. Quantitative real-time PCR (RT-qPCR) was performed using a StepOnePlus Real-Time PCR System (Applied Biosystems, USA). Gene expression levels were normalized to the housekeeping gene B2m.

*Lgr5*-F: 5'-AGC ATG CTT CTG GCA AGA TGT TC-3'; R: 5'-GAC TTA ACG CCC TGC GTT TGA-3', *Sox2*-F: 5'-CTG GAC TGC GAA CTG GAG AAG-3'; R: 5'-TTT GCA CCC CTC CCA ATT C-3', *Shh*-F: 5'-CGG ACC TTC AAG AGC CTT ACC-3'; R: 5'-GCA TAG CAG GAG AGG AAT GC-3', *K8*-F: 5'-TGG AGC AGC AGA ACA AGA TG-3'; R: 5'- CCG CCT AAG GTT GTT GAT GT-3', *Lef1*-F: 5'-ACT GTC AGG CGA CAC TTC CAT G-3'; R: 5'-GTG CTC CTG TTT GAC CTG AGG T-3', *Dvl3*-F: 5'-TGA TGG ACG CAT TGA GCC AGG A-3'; R: 5'-ACA ATC TCC CGA AGG ACT CGG A-3', *Wnt5a*-F: 5'-GGA ACG AAT CCA CGC TAA GGG T-3'; R: 5'-AGC ACG TCT TGA GGC TAC AGG A-3', *Jag1*-F: 5'-TGC GTG GTC AAT GGA GAC TCC T-3'; R: TCG CAC CGA TAC CAG TTG TCT C-3', *Adam10*-F: 5'-TGC ACC TGT GCC AGC TCT GAT G-3'; R: GAT AGT CCG ACC ACT GAA CTG C-3', *Bmp2*-F: 5'-AAC ACC GTG CGC AGC TTC CAT C-3'; R: CGG AAG ATC TGG AGT TCT GCA G-3', *Rictor*-F: 5'-CAG TGT GAG GTC CTT TCC ATC C-3'; R: GCC ATA GAT GCT TGC GAC TGT G-3', *Rps6kb1*-F: 5'-AGG TGG AAC CTC CCT TTA AGC C-3'; R: CCA GAA AGA CCT GGT TGG CAC T-3', *Rps6ka3*-F: 5'-TAA CCG CAG AGG TCA CAC TCA G-3'; R: CTC AGA AAC TGT GGC ATC CCG A-3'; *Pten*-F: 5'-TGA GTT CCC TCA GCC ATT GCC T-3'; R: 5'-GAG GTT TCC TCT GGT CCT GGT A-3', *Gja1*-F: 5'-GGT GAT GAA CAG TCT GCC TTT CG-3'; R: 5'-GTG AGC CAA GTA CAG GAG TGT G-3', *Hdac2*-F: 5'-GTT TTG TCA GCT CTC CAC GGG T-3'; R: 5'-CTT GGC ATG ATG TAG TCC TCC AG-3', *Ogt* -F: 5'-GGC TAT GTG AGT TCT GAC TTC GG-3'; R: 5'-GAT TGG CTT CCG CCA TCA CCT T-3'.

### Cell profile counting

For each tissue section, immunopositive cells were manually quantified by identifying distinct cellular profiles characterized by elongated morphology and clearly defined nuclei within the CVP, specifically along the ducts of VEG.

### Statistical analysis

Data are presented as mean ± standard deviation (SD). Statistical analyses were performed using GraphPad Prism 8 (GraphPad Software, San Diego, CA, USA). Differences between two groups were assessed using unpaired two-tailed Student's *t*-test, whereas comparisons among multiple groups were conducted using one-way analysis of variance (ANOVA) followed by Tukey's multiple comparisons test. A p-value < 0.05 was considered significant. Sample sizes (n) represent the number of biological replicates and are indicated in the figure legends. Statistical details, including the statistical tests used and definitions of error bars, are provided in each figure legend.

## Results

### Lgr5-expressing cells are essential for maintaining the epithelial integrity of the CVP

**Figure 1:**
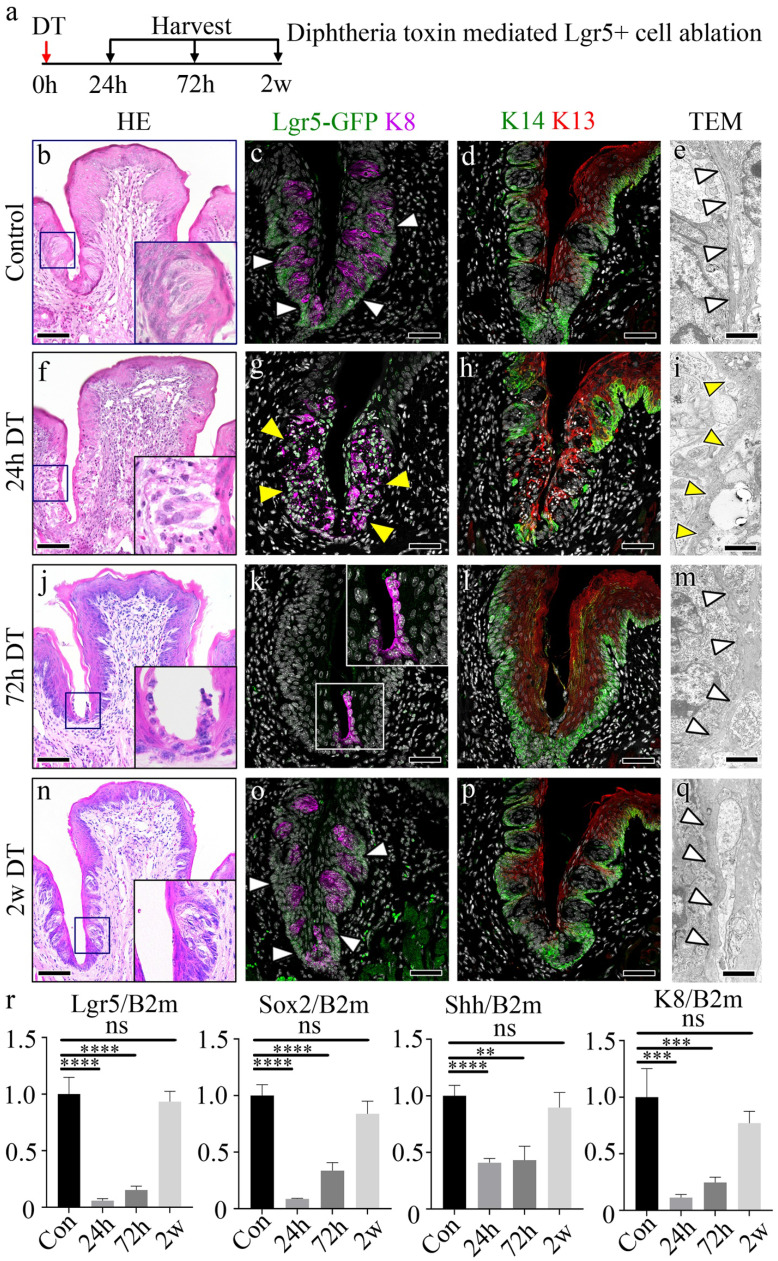
** Characterization of DT-mediated Lgr5 ablation in the CVP. (a)** The experimental timeline illustrates the DT treatment and sacrifice schedule of the mice. **(b)** HE staining shows the CVP trench region with intact taste buds and basement membranes in control mice. **(c)** Lgr5-GFP is detected on the membrane of cells in the deep trench and cells surrounding the taste buds in control mice. K8 is clearly observed in taste buds. **(d)** Distinct populations of K14-expressing basal cells and K13-expressing non-taste epithelial cells are observed on the apical side. **(e)**, TEM micrograph showing a clear and distinct basement membrane in the control CVP (arrowheads). **(f)** HE staining reveals degeneration of the taste-bud-forming region, including the taste bud and basement membrane, which expresses Lgr5+ cells. **(g)** One dose of DT ablated all Lgr5-GFP+ cells at 24 h, as indicated by the lack of GFP signal with disruption of the taste buds and basement membrane. **(h)** Disruption of K14-expressing basal cells and apical K13+ non-taste epithelial cells is observed, indicating the degeneration of basal cells in the taste-bud-forming region. **(i)** TEM micrograph showing the absence of the basement membrane structure in the taste-bud-forming region of the CVP (dotted line). **(j)** HE staining indicates the regenerated morphology of the CVP with the regeneration of basal cells; however, no taste buds are observed. **(k)** Basal CVP trenches are regenerated at 72 h; however, no GFP signal is detected in the CVP. Linearized K8+ cells are observed along the CVP and VEG joint regions in the absence of the taste bud marker K8 in the taste-bud-forming region at 72 h. **(l)** K14 and K13 staining reveals regeneration of basal and non-taste epithelial cells. **(m)** TEM micrograph shows the reappearance of the basement membrane structure 72 h after DT treatment (arrowheads). **(n)** HE staining indicates that CVP morphology with taste buds was recovered, similar to the control CVP. **(o)** Regenerated K8+ taste buds are observed 2 w after DT-induced ablation along with Lgr5-GFP+ cells around the taste buds and in the trench of the CVP. **(p)** K14+ and K13+ cells surrounding the taste buds on the basal and apical sides, respectively, are observed. **(q)** TEM micrograph showing a fully regenerated basement membrane structure, similar to that of the control (arrowheads). **(r)** The expression levels of Lgr5, Sox2, Shh, and K8 are markedly reduced at 24 h after DT treatment. Gradual increases in expression levels of these genes are observed until 2 w after DT treatment. These findings demonstrate that Lgr5+ cells are essential for maintaining CVP epithelial integrity, and their loss leads to rapid degeneration followed by progressive regeneration. Data are presented as mean ± SD. n = 6 biological replicates. Statistical significance is determined using one-way ANOVA followed by Tukey's multiple comparisons test. Scale bar: b,f,j,n - 100 µm, c,d,g,h,k,j,o,p -75 µm, e,i,m,q -1 µm

To assess the role of Lgr5+ stem/progenitor cells in maintaining the CVP epithelium, we performed selective ablation of these cells in Lgr5^DTR^ mice using diphtheria toxin (DT) injection, and the tongues were harvested at 24 hours (h), 72 h, and 2 weeks (w) post-injection (Fig. 1a). Hematoxylin and Eosin (HE) staining of the control revealed a well-formed dome-shaped CVP structure with taste buds (Fig. 1b). Immunohistochemistry revealed well-formed taste buds marked by K8 and Lgr5+ cells in the basal stem/progenitor cells marked with the Lgr5-GFP (Fig. 1c). The presence of Lgr5-expressing cells was further confirmed using RNAscope. Lgr5 was clearly and broadly distributed in the taste-bud-forming and crypt regions of the CVP (Supplementary Fig. 1a). Distinct levels of Keratin 14 (K14), a basal progenitor cell marker, and Keratin 13 (K13), a non-taste-epithelial-cell marker, were observed in the control group (Fig. 1d). Transmission electron microscopy (TEM) revealed a clearly defined basement membrane structure (white arrowheads) in the control group (Fig. 1e). Intact epithelial junctions marked by E-cadherin and p63 positive basal cells were well formed in control. Moreover, proliferative basal cells, as indicated by PCNA, and few or no apoptotic cells were observed (Supplementary Fig. 1e, i). 24 h after DT administration, marked disruption of the epithelial structure, including the basement membrane (yellow arrowheads), was observed, with scattered K8+ taste receptor cells and loss of Lgr5 expression (Fig. 1f-g). K14-expressing basal progenitor cells were lost in the taste-bud-forming region, along with the disruption of K13+ non-taste-epithelial-cells at 24 h after DT treatment (Fig. 1h). TEM analysis at this time point showed complete loss of the basement membrane structure (Fig. 1i). Reduced Lgr5 expression was detected by RNAscope at 24 h after DT injection (Supplementary Fig. 1b). The tight epithelial junctions marked by E-cadherin were disrupted in the taste-bud-forming and crypt regions, and apoptotic cells were observed, with no proliferating cells in the taste-bud-forming region. The basal cell marker p63 was undetectable from the CVP crypt to the taste-bud-forming region (Supplementary Fig. 1f, j). 72h after DT treatment, basal cells and the CVP structure were notably regenerated, lacking taste buds (Fig. 1j). K13+ non-taste-epithelial-cells and K14+ basal cells were localized along the CVP epithelium. However, Lgr5-GFP expressing cells remained undetected at this point, and neither taste buds nor taste receptor cells were observed (Fig. 1k-l). Although K8+ taste receptor cells were not detected in the taste-bud-forming region, K8+ cells were detected from the bottom of the CVP crypt to the furrow region (Fig. 1k). The basement membrane was also regenerated at this stage, as observed in the TEM image (Fig. 1m). Epithelial tight junctions and proliferating basal cells expressing p63 were observed. No apoptotic cells were evident at 72h after DT injection (Supplementary Fig. 1g, k). *Lgr5* mRNA was weakly detected 72 h after DT treatment (Supplementary Fig. 1c). 2 w after DT treatment, the morphology of the CVP resembled that of the control (Fig. 1n). K8+ taste buds were observed in the CVP, and Lgr5-expressing cells were localized to the taste-bud-forming region and the trench of the CVP (Fig. 1o). The expression levels of K14 and K13 were similar to that of the control mice (Fig. 1p). The basement membrane structure also resembled that of the control mice (Fig. 1q). The epithelial tight junctions, basal cells, proliferating basal cells and apoptotic cells were similar to those in the control, along with newly regenerated taste buds (Supplementary Fig. 1h, m). RNAscope analysis further confirmed the restoration of Lgr5 expression (Supplementary Fig. 1d). Reverse transcription quantitative polymerase chain reaction (RT-qPCR) was performed to assess changes in gene expression levels of stem, progenitor, and precursor marker genes, as well as taste receptor cell marker genes. Three time points (24 h, 72 h, and 2 w) after DT treatment were selected to assess gene expression levels during the degeneration and regeneration phases. The stem/progenitor cells (Lgr5), progenitor cells (Sox2), precursor cells (Shh), and taste receptor cells (K8) were evaluated. The results revealed an initial downregulation of these genes at 24 h after DT treatment, followed by a steady increase in gene expression levels 2 w after DT treatment (Fig. 1r). A decrease in the number of proliferative cells was observed at 24 h and an increase in the number of proliferative cells was observed at 72 h after DT treatment compared to the number in the controls. The proliferative cells returned to a number similar to that of the controls 2 w after DT treatment (Supplementary Fig. 1m). Upon DT administration, the Lgr5+ domain within the CVP epithelium undergoes rapid depletion, with extensive cellular degradation observed at 24 h post-DT treatment. By 72 h post-DT, structural restoration of the CVP epithelium is evident, accompanied by reconstitution of the basement membrane. However, the precise cellular origin of the regenerating epithelial population remains unclear, suggesting contributions from adjacent basal progenitors or the transdifferentiation of residual epithelial cells. Notably, by 2 w post-DT, the Lgr5+ domain is re-established, restoring homeostasis within the CVP epithelium (Supplementary Fig. 1n).

### Loss of Lgr5+ cells induces dynamic transcriptional changes in CVP regeneration

**Figure 2:**
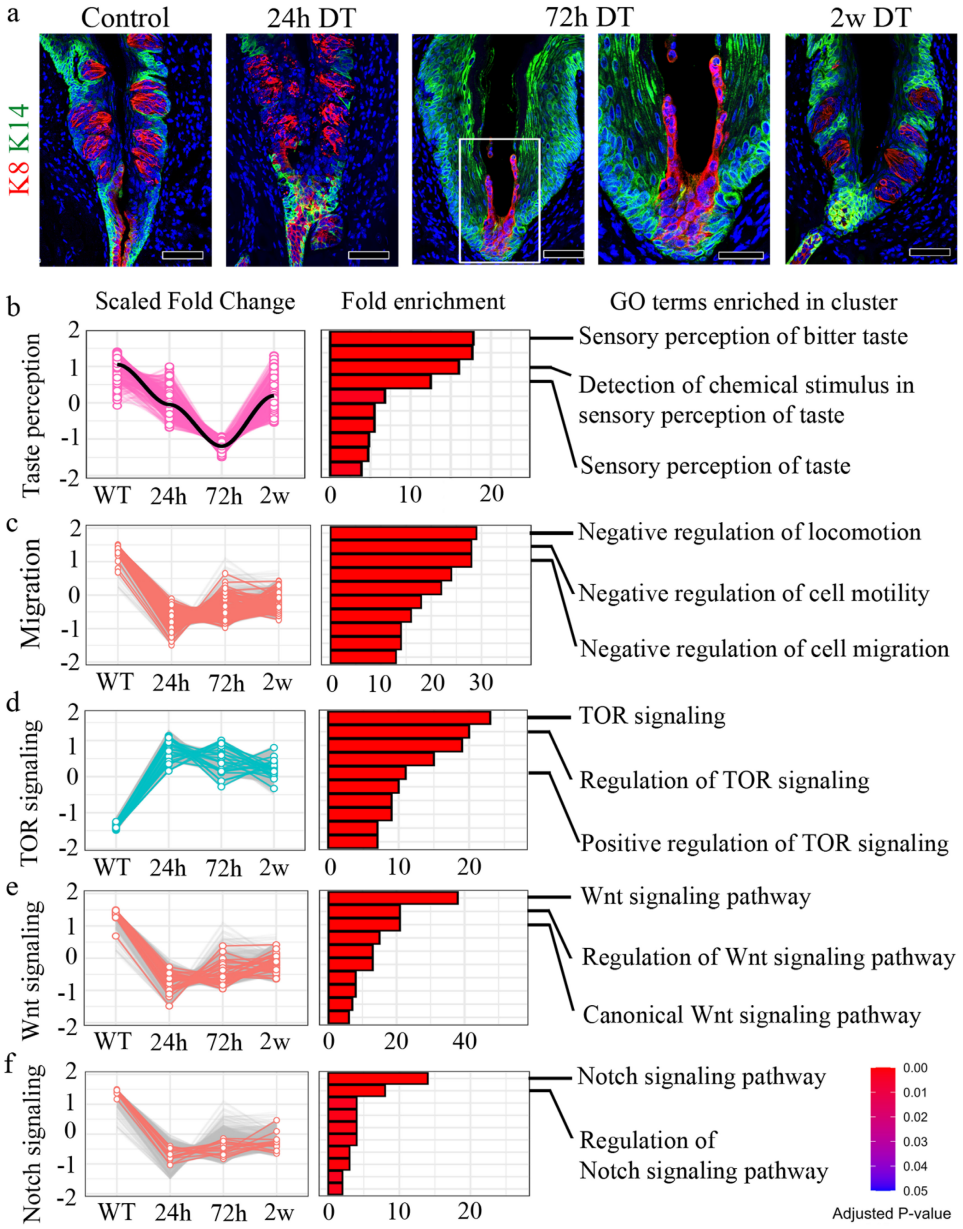
** Dynamic changes in signaling pathways during taste bud regeneration following Lgr5+ cell ablation. (a)** Immunofluorescence staining of CVP at different time points following DT-mediated ablation of Lgr5+ cells. K8 (red) marks differentiated taste bud cells, while K14 (green) labels basal epithelial cells. **(b-f)** Bulk RNA sequencing analysis identifies differentially regulated pathways over time. Scaled fold change expression (left) and Gene Ontology (GO) term enrichment (right) are shown for each cluster. **(b)** Taste perception-related genes are downregulated following Lgr5+ cell ablation and gradually recovered by 2 w. **(c)** Cell migration-related genes are transiently upregulated at 24h and 72h, correlating with tissue damage and repair. **(d)** TOR signaling, a known wound healing regulator, was strongly activated at 24h post-DT treatment and gradually decreased as regeneration progressed. **(e)** Wnt signaling, essential for taste cell turnover and differentiation, is downregulated upon taste bud loss and reactivated by 2 w. **(f)** Notch signaling, which coordinates taste cell differentiation, followed a pattern similar to that of Wnt signaling, showing initial suppression followed by upregulation during regeneration. These findings demonstrate that Lgr5+ cell ablation induces dynamic, stage-specific changes in key signaling pathways, characterized by early activation of cell migration and wound healing responses, followed by coordinated reactivation of differentiation pathways during taste bud regeneration. Color intensity in the bar graphs represents adjusted p-values. Scale bars, 75 µm.

To investigate the dynamic epithelial changes following Lgr5+ cell ablation, we examined CVP morphology over time using immunofluorescence staining for K8, a marker of differentiated taste bud cells, and K14, which labels basal progenitor cells (Fig. 2a). In control tissues, K8+ taste bud cells were organized within the CVP, surrounded by K14+ basal progenitor cells. At 24 h post-DT administration, we observed a reduction in K8+ taste receptor cells with disruption of taste bud structure, and a decrease in K14+ basal progenitor cells in the CVP epithelium. By 72 h, the loss of Lgr5+ cells resulted in a striking reorganization of the CVP, characterized by the elongation and apparent migration of K8+ cells towards the basal region. At 2 w post-ablation, the CVP began to regain structural integrity, with partial restoration of K8+ cell organization and taste bud structure (Fig. 2a).

To investigate the molecular mechanisms underlying CVP regeneration following Lgr5+ cell depletion, we performed bulk RNA sequencing (RNA-seq) at multiple time points (0h, 24h, 72h, and 2 w post-DT treatment) and clustered differentially expressed genes (DEGs) into functional categories (Fig. 2b-f). Gene Ontology (GO) term enrichment analysis revealed distinct temporal transcriptional programs associated with taste perception, cell migration, and key signaling pathways, including TOR, Wnt, and Notch signaling.

A subset of DEGs was associated with taste perception, including genes involved in taste detection and chemical stimulus responses (Fig. 2b). These genes were downregulated at 24h post-DT treatment. They reached their lowest expression levels at 72h, coinciding with the peak of Lgr5+ progenitor cell loss. However, by 2 w, their expression partially recovered, suggesting an adaptive response to progenitor cell depletion.

Genes involved in negative regulation of cell motility and migration showed a distinct pattern of downregulation at 72h post-DT treatment (Fig. 2c). GO terms related to the negative regulation of locomotion, cell motility and migration were significantly enriched at this time point, suggesting that non-progenitor epithelial cells, including VEG ductal cells, may compensate for the loss of Lgr5+ cells by modulating their migratory properties.

Previous studies have established TOR signaling as a critical regulator of wound healing 32. Consistent with this, our analysis revealed an upregulation of TOR pathway genes at 24h post-DT treatment, coinciding with the peak of tissue damage observed in CVP following Lgr5+ cell ablation (Fig. 2d). GO term enrichment analysis identified a significant increase in genes associated with TOR pathway activation, its regulation, and positive modulation of TOR signaling, that suggests that TOR pathway-associated genes are transcriptionally upregulated during the early regenerative phase by promoting cellular repair.

Wnt signaling is a well-established regulator of taste cell turnover, primarily by controlling progenitor cell differentiation 33,34. In our study, DT-mediated ablation of Lgr5+ cells led to a progressive loss of taste buds, with noticeable degeneration at 24h post-treatment and a complete absence of taste bud structures by 72h. However, by 2 w, taste buds were regenerated, suggesting the activation of compensatory mechanisms to restore taste cell homeostasis. Consistent with this dynamic process, Wnt signaling pathway genes were initially downregulated at 24h post-DT treatment, correlating with the loss of taste bud structures (Fig. 2e). However, at 2 w, Wnt signaling components showed significant upregulation, as revealed by GO term enrichment for canonical Wnt pathway activation and its regulation. These results indicate that Wnt pathway-associated genes are transcriptionally upregulated during regeneration, suggesting a potential role in taste cell differentiation of new taste cells from surviving progenitor populations, thereby facilitating taste bud regeneration.

Notch signaling is a well-established regulator of taste cell differentiation, working in coordination with Wnt signaling to maintain taste bud homeostasis 33, 34. Notch signaling pathway genes were initially downregulated at 24h post-DT treatment, coinciding with the loss of taste bud structures (Fig. 2f). However, by 2 w, Notch pathway genes exhibited significant upregulation, as indicated by GO term enrichment for Notch pathway activation and regulation. These results suggest that Notch pathway-associated genes show increased expression during regeneration, consistent with a role in taste cell differentiation of newly forming taste cells, ensuring proper taste bud re-establishment. GO enrichment analysis identified biological processes associated with epithelial morphogenesis, migration, and wound healing (Fig. 2b-f). The individual genes contributing to each GO term are listed in Supplementary Table 1.

To validate the signaling dynamics identified by bulk RNA-seq, we performed RT-qPCR and immunofluorescence (IF) analyses at sequential intervals following Lgr5⁺ cell ablation. RT-qPCR revealed that migration-inhibitory genes were markedly downregulated at 24 h post-DT administration and remained suppressed throughout the early regenerative phase before showing partial recovery at later stages. Similarly, Wnt- and Notch-associated transcripts were sharply attenuated at 24 h but were progressively re-expressed during the regenerative process. In contrast, TOR-associated genes exhibited an acute induction following injury (Supplementary Fig. 2a). These temporal transcriptomic shifts were concordant with protein-level changes observed via IF. Immunoreactivity for HDAC2, β-CATENIN, and HES1 was significantly diminished following DT treatment and gradually recovered as the CVP regenerated. Conversely, p-p70S6K expression was transiently upregulated during the early post-injury phase, signaling rapid TOR pathway activation. Collectively, these data corroborate our transcriptomic findings and demonstrate that CVP regeneration following Lgr5⁺ cell loss is characterized by the transient suppression of migration-inhibitory, Wnt, and Notch signaling, alongside the early activation of the TOR pathway (Supplementary Fig. 2b).

### K14+ cells from Von Ebner's duct promote regeneration of the CVP crypt

**Figure 3:**
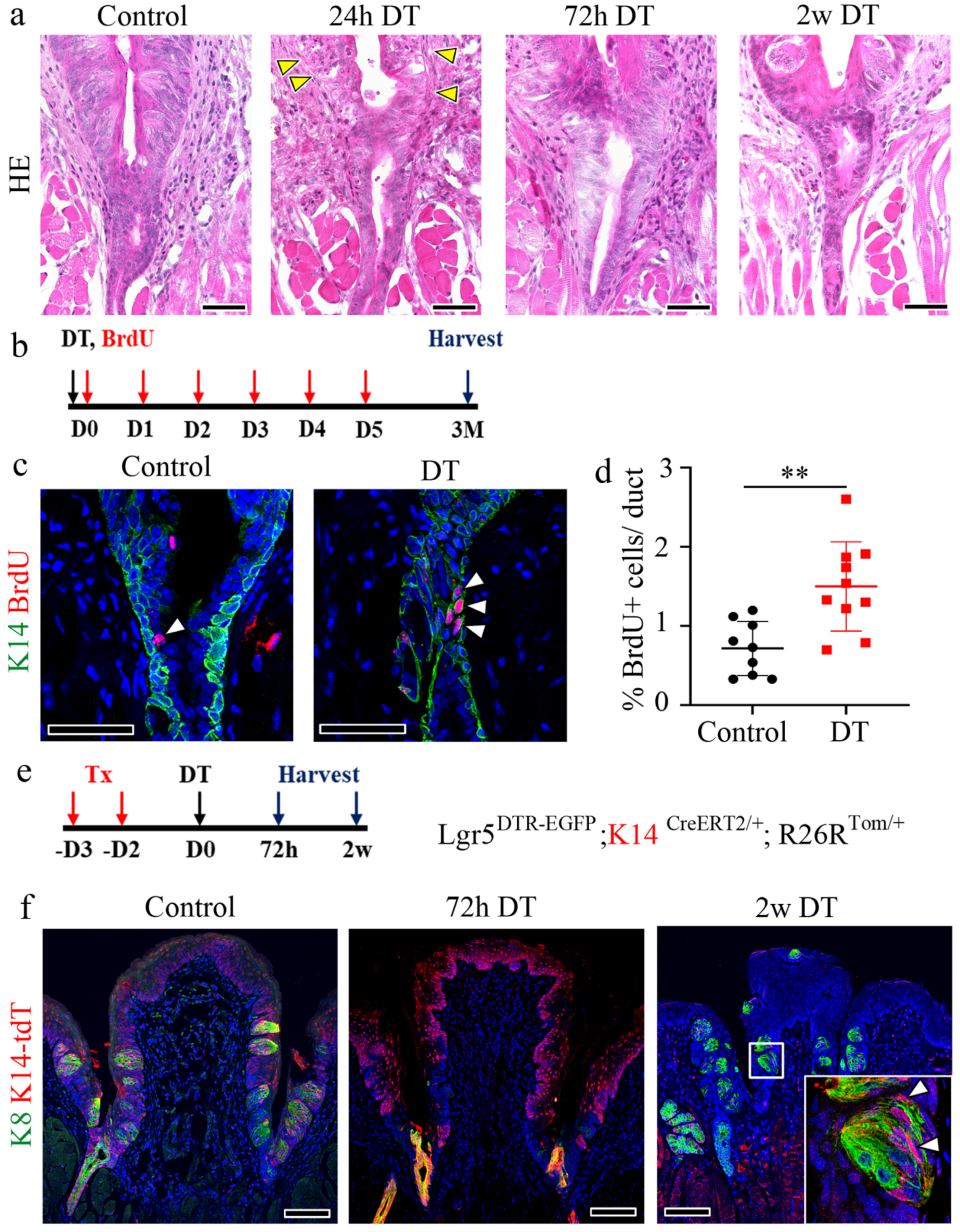
** Cells derived from K14+ cells are label-retaining cells and regenerate the CVP. (a)** HE staining of the VEG duct at different time points after DT treatment, indicating that ductal cells are not affected by Lgr5 ablation. Yellow arrowheads indicate basal cell degeneration of the CVP at 24 h. **(b)** Experimental timeline for BrdU injection and CVP harvest is illustrated to confirm the label-retaining cells in the VEG. **(c)** BrdU+ label-retaining cells are found in the basal layer of the VEG co-localized with K14 (arrowhead), both in the control and the DT-injected CVP at 3 months after injury. **(d)** Quantitative analysis indicating the number of BrdU+ label-retaining cells in the control and DT-injected CVP. The DT-injected CVP shows an increase in the number of BrdU+ label-retaining cells. Data are presented as mean ± SD. n = 6 biological replicates. Statistical significance was determined using unpaired two-tailed t-test. **(e)** Lineage tracing results of Lgr5^DTR-EGFP^; K14^CreERT2/+^; R26R^Tom/+^ mice to identify K14-derived cells after Lgr5 ablation. **(f)** In control mice, K14-derived cells can be observed in the taste buds and surrounding perigemmal cells 3 days after tamoxifen injection. At 72 h after DT treatment, K8+ taste receptor cells are not detected in the entire CVP epithelium. Most of the CVP cells were K14-derived tdT+ cells, and most of those around the junction region of the CVP crypt and VEG duct show K8 expression. 2 w after DT treatment, the taste buds and basal cells express K14-derived tdT+, suggesting the involvement of K14+ cells in CVP regeneration following Lgr5 ablation. These findings demonstrate that K14+ ductal cells in the VEG function as label-retaining, injury-responsive progenitors that contribute to CVP regeneration following Lgr5+ cell ablation. Scale bar: a, 50 µm; c, 75µm; f, 100 µm

To confirm the expression of Lgr5 in the VEG duct, we used Lgr5^CreERT2/+^; Rosa26^Tom/+^ reporter mice. Immunofluorescent staining demonstrated that Lgr5+ stem cell-derived cells were not detected in the VEG duct, but were clearly observed in the CVP, including the taste buds 6 w after tamoxifen treatment (Supplementary Fig. 3a, b). This finding confirmed that Lgr5+ cells do not contribute to ductal cell homeostasis. Consistent with this, as Lgr5+ cells did not contribute to the ductal cells, VEG ducts remained unaffected following DT treatment, as observed by HE staining (Fig. 3a). Immunofluorescence staining was performed to assess the distribution of K14 and K8 within the VEG region. K14 serves as a marker for basal progenitor cells, whereas K8, although widely used to label taste receptor cells within gustatory tissues, is also expressed in non-gustatory epithelium. As the VEG does not contain taste receptor cells, K8 and K14 expression in this context is more appropriately interpreted as marking VEG ductal epithelial cells rather than cells of gustatory lineage. A two-layered ductal structure was observed, with K14+ cells in the basal layer and K8+ cells in the luminal layer of the VEG duct (Supplementary Fig. 3c). These findings indicated that K14 positive; Lgr5 negative cells represent VEG ductal cells, but not CVP epithelial cells in the taste bud-forming region. Quiescent stem cells are activated exclusively in response to injury 35, 36. To identify this slow-cycling, injury-responsive progenitor population in the VEG, 5-bromo-2-deoxyuridine (BrdU) was injected into mice following DT injection and the mice were chased for 3 months (Fig. 3b). Data analysis revealed the presence of label-retaining cells in the VEG ducts expressing K14 after DT treatment (Fig. 3c). An increase in label-retaining cells was observed after DT-mediated Lgr5 ablation (Fig. 3d). These data suggest the presence of a slow-cycling, injury-responsive progenitor population in the VEG, expressing basal/progenitor markers that are activated during the process of regeneration in the CVP. Building on a previous report showing that Sox10-Cre-labeled tdTomato+ (tdT+) cells were broadly distributed throughout the VEG duct, with a subset also detected within taste buds 12. Given the distinct basal-luminal organization of the VEG duct, we examined whether basal K14+ cells were activated to compensate for the loss of Lgr5+ stem/progenitor cells in the CVP. For lineage tracing, Lgr5^DTR-EGFP^; K14^CreERT2/+^; R26R^Tom/+^ mice received tamoxifen 3 days before DT injection (Fig. 3e). Upon the ablation of Lgr5-expressing cells, the progeny derived from K14+ cells in the VEG duct were mobilized to regenerate the CVP epithelium. In the control group, K14-derived tdT+ cells were observed in the taste buds, as well as in the perigemmal and basal cells of the CVP epithelium. At 72 h post-DT-treatment, K14-derived tdT+ cells were strongly detected in the crypt region where the ducts of the VEG joined the CVP, with scattered cells along the trench of the CVP. After 2 w, tdT-labelled cells were found inside the regenerated taste buds. Of note, some tdT+ cells inside the taste buds did not colocalize with the taste receptor cell marker K8 (Fig. 3f). To assess whether label-retaining cells can re-enter the cell cycle, we established a double-injury model. A second DT injection was given 2 w after the first, when the CVP had regenerated, and tissues were collected 2 w later. BrdU was administered six times after the first DT injection, and 5-iodo-2′-deoxyuridine (IdU) was co-injected with the second DT (Supplementary Fig. 3d). Immunohistochemistry revealed that a population of cells in the VEG incorporated both BrdU and IdU, indicating that BrdU-label-retaining cells re-entered the cell cycle during the regeneration phase (Supplementary Fig. 3e). HE staining revealed that the BrdU/IdU+ cells were the basal cells of the VEG duct (Supplementary Fig. 3f). These data suggest that label-retaining cells in the VEG may function as a reserve or injury-responsive progenitor population that are reversibly activated upon injury.

### Sox10+ basal cells in the VEG duct exhibit quiescent, hillock-like characteristics and the potential for migration during regeneration

**Figure 4:**
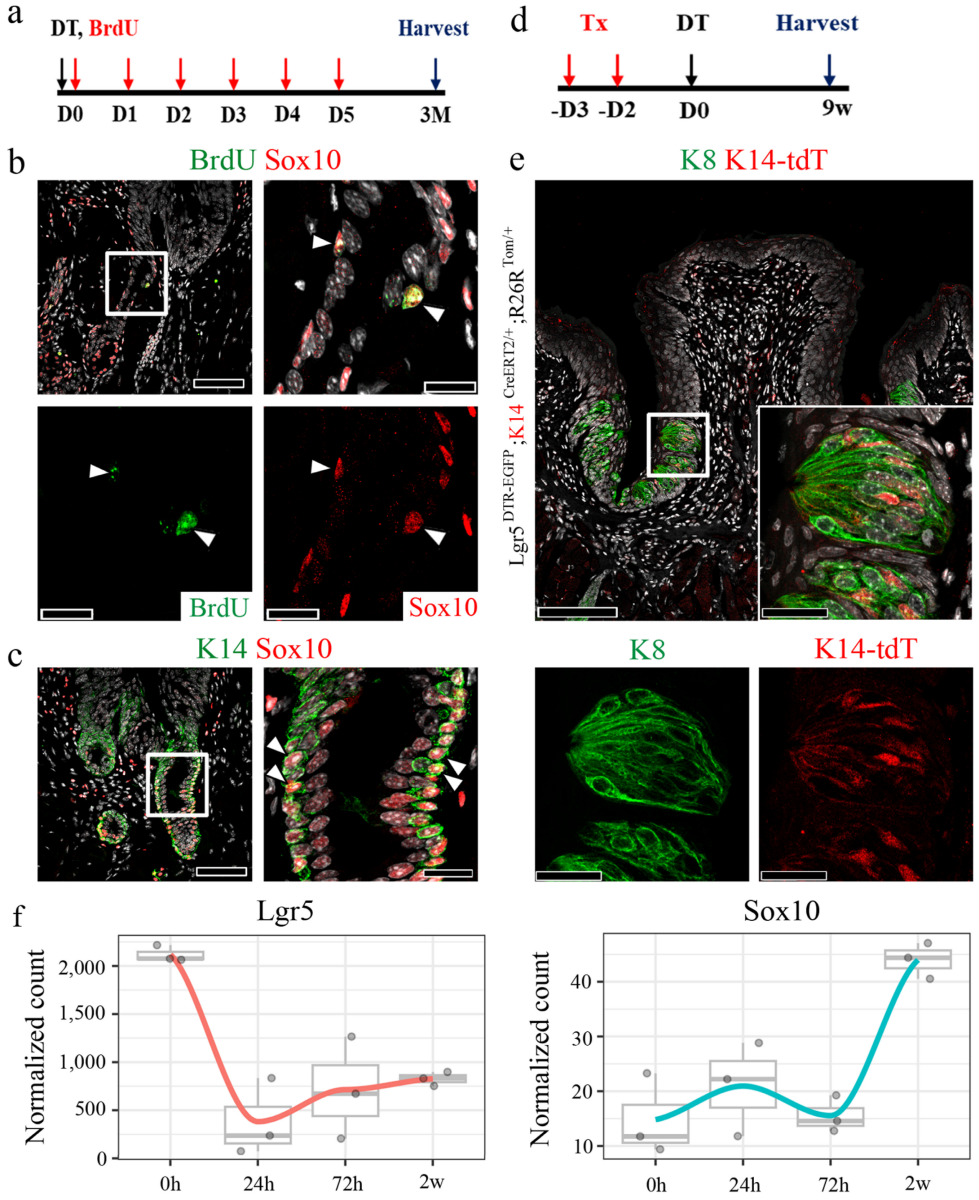
** Existence of quiescent, Sox10+ basal cells in the VEG duct. (a)** Experimental timeline of BrdU injection and CVP harvesting. **(b**,**c)** Immunofluorescence data showing ductal cells expressing Sox10 co-localizing with BrdU+ label-retaining cells (arrowhead) and K14+ basal cells.** (d)** Schematic timeline indicating long-term tracing of K14+ cell-derived tdT+ cells. **(e)** tdT+ taste receptor cells are observed in taste buds after 9 w of tracing.** (f)** Bulk RNA-seq analysis is performed to assess transcriptional changes upon DT-induced ablation of Lgr5+ cells. Lgr5 expression exhibits a sharp decline, reaching its nadir at 24 h post-ablation, followed by a gradual recovery over the subsequent 2 w. Sox10 expression, a marker of VEG ductal cells, displays a progressive increase, peaking at 2 w post-ablation. These findings demonstrate that Sox10+ basal cells in the VEG duct represent a quiescent, label-retaining progenitor population that is activated in response to injury and may contribute to CVP regeneration following Lgr5+ cell ablation. Data are presented as mean ± SD. n = 3 independent experiments. Statistical significance was determined using one-way ANOVA. Scale bar: b,c,e, 100 µm; b,c high magnification, 75 µm, e high magnification, 25 µm

A recent study suggested the presence of Sox10+ cells in the VEG duct and their role in the homeostatic maintenance of taste buds 13. To investigate whether Sox10+ cells constitute the pool of label-retaining cells, immunostaining for BrdU and Sox10 was performed in CVP tissue following 3 months of chasing with BrdU (Fig. 4a). Of note, a subset of Sox10+ cells co-localized with BrdU+ cells in the basal layer (Fig. 4b). This result was further validated by co-staining for Sox10 and K14, markers of ductal and basal cells, respectively. These findings indicated that Sox10+ cells constitute a population of basal cells in the VEG duct that cycle slowly, remain relatively quiescent in homeostasis, and are activated only during injury (Fig. 4c). Furthermore, long-term lineage tracing revealed the presence of K14+ progeny even 9 w after Lgr5 ablation, suggesting that K14+ cells may contribute to the regeneration of Lgr5+ cells, as well as the long-term maintenance of taste buds (Fig. 4d, e).

To investigate whether the loss of Lgr5+ cells led to the involvement of Sox10+ VEG ductal cells in epithelial regeneration, bulk RNA-seq analysis was performed. Upon DT-induced ablation of Lgr5+ cells, the expression of Lgr5 markedly declined, reaching its lowest level at 24 h post-ablation, followed by a gradual recovery over the subsequent 2 w. Concomitantly, Sox10 expression, a marker of VEG ductal cells, exhibited a progressive increase, peaking at 2 w post-ablation (Fig. 4f). This dynamic regulation suggests a compensatory response wherein Sox10+ VEG ductal cells may contribute to the regenerative process following Lgr5+ cell depletion.

To determine whether Sox10+ cells involved in regeneration originate from intra-bud populations or surrounding epithelial regions, we mapped their spatio-temporal distribution following Lgr5+ cell ablation (Supplementary Fig. 4a-h). Under control conditions, Sox10+ cells were detected within the intra-bud compartment, intermingled with K8+ taste receptor cells (Supplementary Fig. 4a, b). At 24 h post-DT treatment, the degeneration of the taste buds was accompanied by a marked loss of these intra-bud Sox10+ cells, reflecting the overall disruption of epithelial integrity (Supplementary Fig. 4c, d). While some Sox10+ cells remained detectable in the underlying mesenchyme (likely representing neural/glial elements), the epithelial Sox10+ population was largely depleted. Crucially, by 72 h post-ablation, a new population of Sox10+ cells emerged, localized predominantly within the CVP crypt epithelium adjacent to the VEG ductal interface (Supplementary Fig. 4e, f). By 2 weeks after DT treatment, Sox10+ cells were again detected within the regenerated taste bud region together with K8+ cells, consistent with re-establishment of epithelial structure (Supplementary Fig. 4g, h). These dynamic shifts, specifically the initial loss of intra-bud Sox10+ cells followed by their emergence at the ductal junction, support the conclusion that VEG duct-associated Sox10+ cells, rather than a surviving intra-bud niche, drive CVP epithelial regeneration following Lgr5+ cell loss.

### Luminal cells from the duct migrate and regenerate the CVP

**Figure 5:**
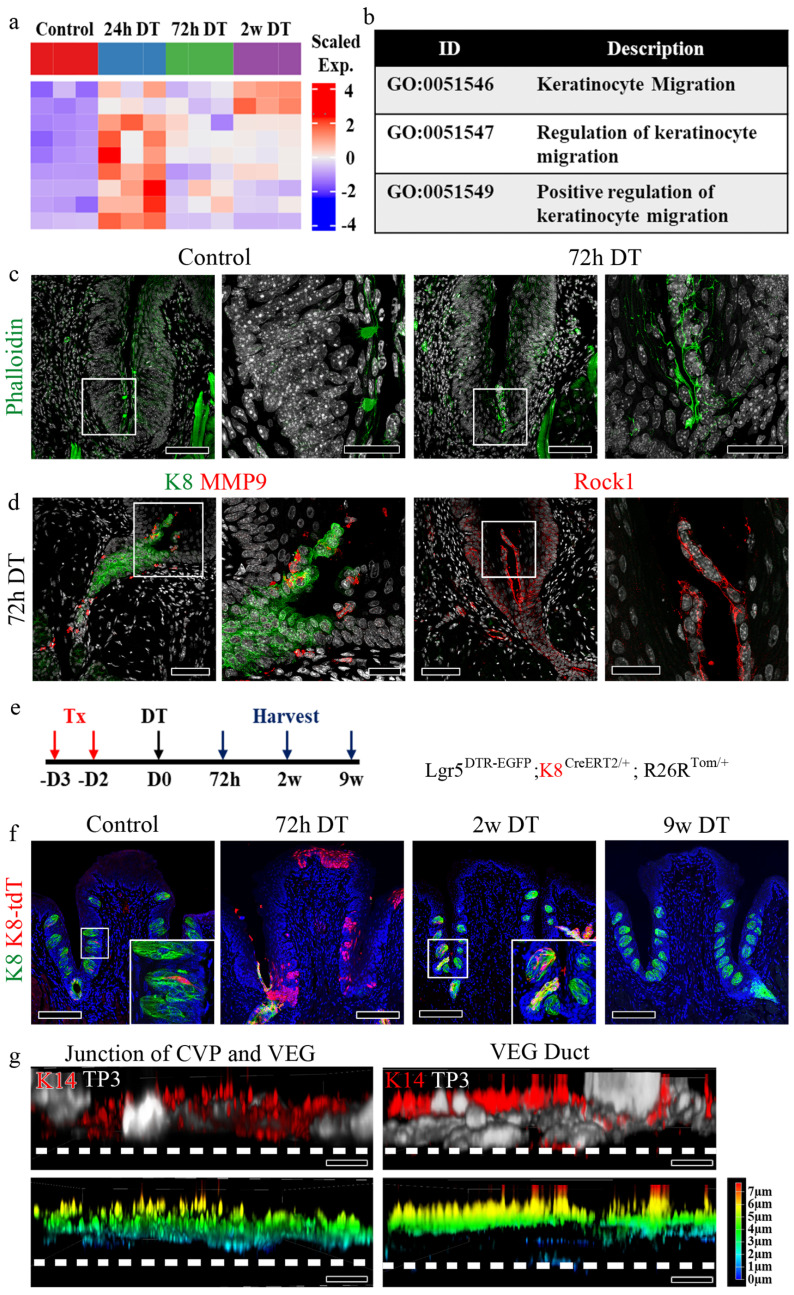
** K8+ luminal cells migrate to the CVP and contribute to taste bud regeneration. (a)** Immunofluorescence staining indicating the expression of K14 and K8 in cells in CVP crypts at different time points after DT injection. After 72 h, luminal K8+ cells migrate into the CVP groove. **(b)** Heat map showing the expression levels of migration related genes at each time point. **(c)** Table indicating Gene Ontology terms with descriptions related to keratinocyte migration. **(d)** Phalloidin staining showing the presence of stress fibers during keratinocyte migration. In control mice, phalloidin staining is observed in the taste pore; however, at 72 h, the luminal cells arising from the VEG duct show strong phalloidin staining, indicating the formation of stress fibers during migration. **(e)** Expression of MMP9 and Rock1 in luminal cells further confirms the migratory behavior of these cells.** (f)** Lineage tracing of Lgr5^DTR-EGFP^; K8^CreERT2/+^; R26R^Tom/+^ mouse is confirmed to identify cells derived from K8+ cells after Lgr5 ablation. Few K8-derived tdT+ cells were detected in the taste buds of the control samples. K8-derived tdT+ cells are observed at the junction of the CVP crypt and the VEG duct 72 h after DT treatment. tdT+ taste receptor cells are observed in both regenerated taste buds and the VEG duct 2 w after DT treatment. K8-derived tdT+ cells completely disappear after 9 w of tracing, not only on taste buds but also on VEG ducts. **(g)** Confocal image of K14+ basal cells from VEG ductal basal cells that do not form contact with the basement membrane compared to the junction of CVP and VEG, as indicated by immunofluorescence and depth coding analysis. These findings demonstrate that K8+ luminal cells from the VEG duct actively migrate into the CVP following Lgr5+ cell ablation and transiently contribute to taste bud regeneration before the restoration of Lgr5+ stem cell-mediated homeostasis. Scale bar: c,d 75 µm, c,d high magnification, 25 µm, f 100 µm; g 5 µm

To investigate the differences in gene expression patterns between the control and Lgr5-ablated CVP, bulk RNA-seq was performed, and transcriptomic changes in cell migration were analyzed (Fig. 5a). Gene Ontology analysis comparing the control and different time points following Lgr5 ablation identified genes that were significantly upregulated during keratinocyte migration (Fig. 5b). To validate the bulk RNA-seq data, we performed immunostaining for various cell migration markers. To identify stress fibers formed during migration, Phalloidin staining was performed in control samples and samples treated with DT for 72 h. In the control, Phalloidin staining was restricted to the taste pore, whereas in the 72 h DT, it was observed in the migrating luminal cells, confirming their migratory behavior (Fig. 5c). Further validation by immunostaining for matrix metalloproteinase-9 (MMP9) and Rock1 showed positive signals in migrating luminal cells, corroborating their migratory behavior (Fig. 5d). Sox10 immunostaining further confirmed that the migrating cells in the groove originated from ductal luminal cells, which was consistent with observations in the control at 72 h after DT treatment (Supplementary Fig. 5a). Additionally, immunostaining with the proliferation marker PCNA revealed increased proliferation of ductal cells at 72 h, except in luminal cells, which did not express the proliferation marker (Supplementary Fig. 5b). Of note, migratory luminal cells expressed Pkp4 and Zo-1, which are markers of desmosomes and apical tight junctions, respectively (Supplementary Fig. 5c). The schematic figure illustrates the distinct protein localization in K8 and K14+ cells during CVP regeneration (Supplementary Fig. 5d). Given that the Sox10-Cre lineage labelled both basal (K14⁺) and luminal (K8⁺) compartments of the VEG duct 12, and K14-CreERT2 tracing revealed the mobilization of basal ductal cells during CVP regeneration, it remained unclear whether the luminal population also contributes to this process. To address this, we generated Lgr5^DTR-EGFP^; K8^CreERT2/+^; R26R^Tom/+^ mice to trace K8+ luminal cells and their progeny following Lgr5+ cell ablation. Tamoxifen was administered according to the protocol used in previous experiments, and tissues were harvested at various time points (Fig. 5e). In the control group, a few tdT+ cells derived from K8+ cells were observed only inside the taste buds. At 72 h post-DT-treatment, K8-derived tdT+ cells were detected in the crypt region, with a few cells scattered along the trench of the CVP. After 2 w, tdT+ cells were found within the regenerated taste buds. Furthermore, lineage tracing at week 9 revealed that tdT+ cells derived from K8+ cells were not detected in the CVP taste buds, indicating that K8+ cells derived from the VEG duct contributed to the initial stages of CVP regeneration following injury. Subsequently, traditional taste bud maintenance mechanisms were restored, as Lgr5 expression was recovered (Fig. 5f). In the airway epithelium, a unique population of cells, termed hillock cells, has been reported to be injury-resistant and to participate in the regeneration of the airway epithelium after injury 37. A unique feature of these cells is that they are squamous epithelial cells that do not contact the basement membrane. To investigate whether basal cells from the duct represent a similar population, K14+ basal cells from the duct and CVP were imaged. Tissue depth analysis revealed that the K14+ basal cells of the CVP contacted the basement membrane. In contrast, the K14+ basal cells in the VEG duct did not, as did the hillock cells (Fig. 5g). These data indicate that ductal basal cells are not attached to the basement membrane and may migrate into the CVP during homeostasis and regeneration.

### VEG ductal cells as a source of Sox10+ taste bud progenitors

**Figure 6:**
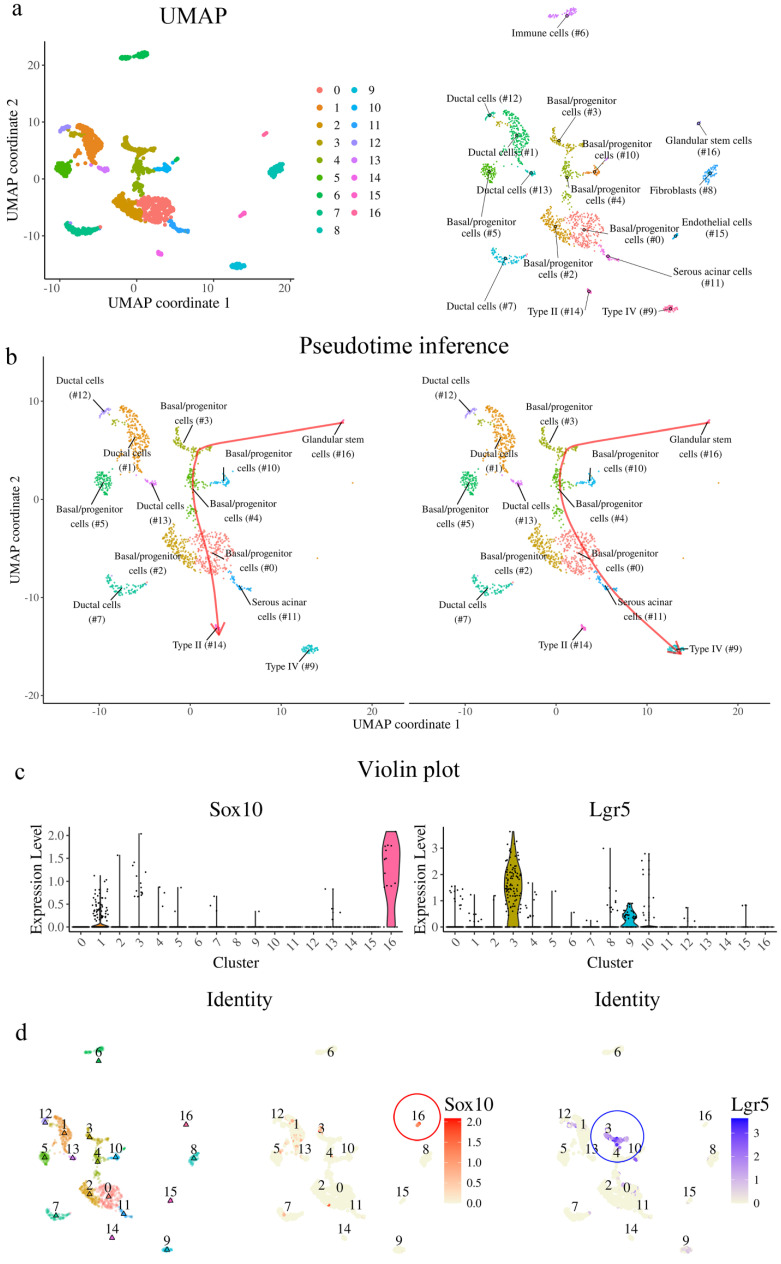
** Single-cell RNA sequencing reveals VEG ductal cells as a Sox10+ progenitor source for taste bud regeneration. (a)** UMAP plot of scRNA-seq data, showing 17 distinct cellular clusters in the CVP and VEG. Each color represents a different cluster. **(b)** Pseudotime-based lineage tracing analysis indicates a differentiation trajectory originating from Sox10+ VEG ductal cells, transitioning through Lgr5+ stem cells, and further differentiating into different types of taste receptor cells. **(c)** Violin plots displaying expression levels of Sox10 and Lgr5 across different clusters. Sox10 expression is highly enriched in cluster 16, suggesting that these cells are VEG ductal cells. Lgr5+ stem/progenitor cells occupy distinct transitional states. **(d)** Feature plots mapping Sox10 and Lgr5 expression onto the UMAP clustering, confirming their sequential distribution along the inferred differentiation trajectory. These findings suggest that Sox10+ VEG ductal cells represent a distinct progenitor population positioned upstream in the differentiation trajectory and may contribute to the generation of Lgr5+ stem/progenitor cells and subsequent taste receptor cell lineages.

To further characterize the cellular composition of VEG and its potential contribution to taste bud regeneration, we re-analyzed single-cell RNA sequencing (scRNA-seq) data from Yu et al., 2024 13. We identified 17 distinct cellular clusters within the tissue, as visualized by UMAP projection (Fig. 6a). Pseudotime trajectory inference further elucidated the differentiation dynamics within this cellular landscape (Fig. 6b). The analysis suggested a continuum in which Sox10+ cells are highly enriched in cluster 16, which is annotated as a “glandular stem cell” cluster in the original dataset, followed by progression through Lgr5+ basal progenitor populations and differentiation into taste receptor cell types. Consistent with this trajectory, violin plot analysis demonstrated that Sox10 expression was predominantly enriched in cluster 16, whereas Lgr5 expression was concentrated in cluster 3, basal progenitor populations (Fig. 6c). Spatial mapping of gene expression confirmed these findings, with Sox10 highly localized to cluster 16 and Lgr5 marking basal progenitors in cluster 3 (Fig. 6d). Analysis of SOX family gene expression revealed that Sox10 expression was highly restricted to cluster 16, whereas Sox2 and Sox9 were distributed across distinct clusters (Supplementary Fig. 6a). Notably, the Sox10-enriched cluster exhibited low levels of Sox2 and Sox9 expression (Supplementary Fig. 6b). Pseudotime analysis showed that Sox10 expression was predominant at early stages of the trajectory, while Sox2 and Sox9 expression increased at later stages (Supplementary Fig. 6c). Importantly, further analysis of marker gene expression across clusters demonstrated that the Sox10-enriched population exhibited a distinct transcriptional profile, characterized by low expression of Sox2 and Sox9, minimal overlap with Krt14-expressing basal progenitor populations, and limited expression of Krt8-associated differentiated epithelial markers (Supplementary Fig. 6d). Collectively, these data support a model in which Sox10+ cells, enriched in the cluster annotated as “glandular stem cells” represent a distinct epithelial population that may be involved in the regenerative process following injury.

### The ablation of Lgr5-expressing cells precludes organoid formation

**Figure 7:**
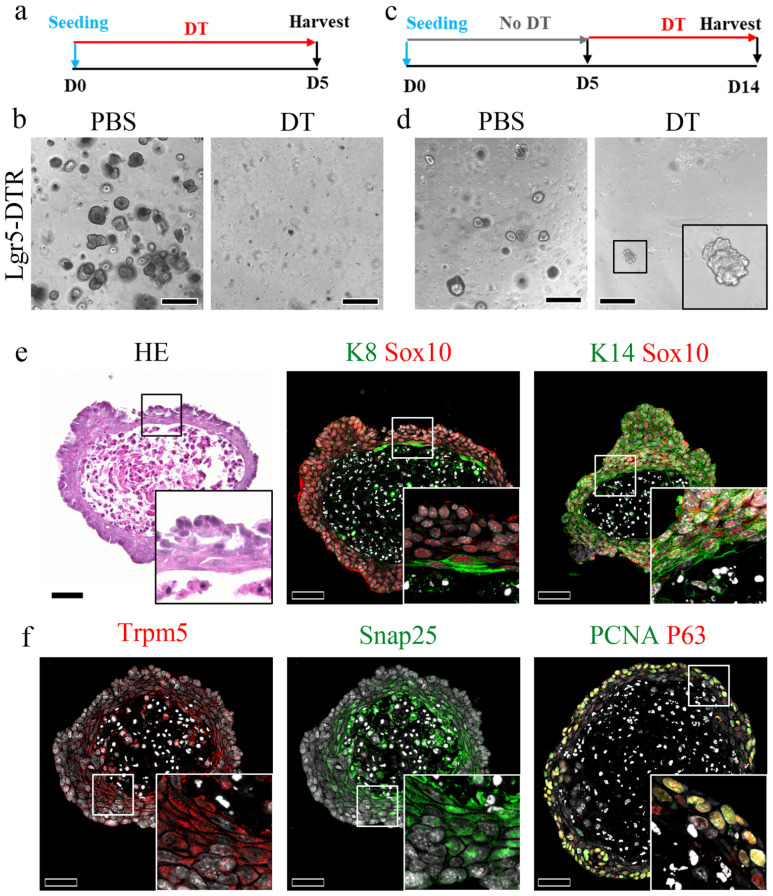
** Organoid formation after ablation of Lgr5 in CVP cells. (a**,**c)** Experimental timeline. **(b)** Well-formed taste bud organoids are observed in PBS-treated wells; however, no organoids are observed in DT-treated wells when DT is added on day 0. **(d)** PBS-treated wells show well-formed organoids, and the addition of DT after 5 days of organoid formation resulted in a few DT-resistant organoids. **(e)** DT-resistant organoids showing a keratinized core surrounded by K8 -and K14-expressing cells representing taste receptor cells and basal cells, respectively, co-localizing with Sox10, indicating their ductal origin. **(f)** DT-resistant organoids also express Trpm5 (Type II) and Snap25 (Type III), taste receptor markers. These organoids also contain P63-expressing basal cells. These findings demonstrate that Lgr5+ stem cells are essential for organoid initiation, while duct-derived epithelial cells can support the survival and differentiation of organoids under Lgr5-depleted conditions. Scale bar: b,d 500 µm; e,f 50 µm

To investigate the contribution of other stem cell populations to the CVP, an organoid formation assay was performed, as only stem/progenitor cells can form organoids. To determine whether Lgr5+ cells are required to initiate organoids, CVP tissue from Lgr5^DTR^ mice was minced into single cells and seeded in the presence or absence of media containing DT for 5 days (Fig. 7a). Numerous organoids formed well in phosphate-buffered saline (PBS). In contrast, no organoids were observed in the DT-treated wells (Fig. 7b). Organoids from Lgr5^DTR^ mice were established for 5 days without DT treatment and then cultured in the presence of DT from days 5 to 14 (Fig. 7c). Few organoids survived DT treatment and were observed until day 14 (Fig. 7d). We subsequently harvested and sectioned DT-resistant organoids to examine the presence of various taste receptor cell markers. HE staining showed that the organoids exhibited a keratinized core, which is a characteristic feature of Matrigel-cultured taste bud organoids. Immunohistochemical analysis demonstrated that these organoids contained K8+ taste receptor cells and K14+ basal cells, both of which co-localized with Sox10, suggesting an association with Sox10+ cells (Fig. 7e). Additionally, these organoids expressed Trpm5 and Snap25, markers of type II and type III taste receptor cells, respectively, indicating their capacity to differentiate into mature taste receptor cells. Furthermore, the organoids contained PCNA and P63-positive basal proliferating cells (Fig. 7f). These findings suggest that Sox10+ epithelial cells that emerge during organoid culture contribute to the survival of organoids containing differentiated taste receptor cell markers under conditions of Lgr5+ cell depletion.

## Discussion

This study provides important insights into the role of Lgr5-expressing stem/progenitor cells in maintaining the structural integrity of the CVP and highlights the compensatory mechanisms that occur following their removal. Our findings demonstrate that Lgr5+ cells are essential for epithelial homeostasis and organoid initiation, and that their ablation leads to rapid disruption of the basement membrane and taste bud architecture, followed by activation of alternative regenerative mechanisms, consistent with previous reports on epithelial stem cell dependency in tissue maintenance 4, 7.

In the absence of Lgr5+ stem cells, we observed rapid epithelial reconstruction within 72 h, preceding the re-establishment of the Lgr5+ compartment. This early recovery phase is characterized by the restoration of basal cells and basement membrane integrity, indicating the presence of compensatory cellular sources independent of canonical Lgr5+ stem cells. Similar compensatory responses have been described in intestinal and hair follicle systems, where non-Lgr5 populations acquire stem-like properties following injury 4, 5.

Our data identify VEG-derived ductal cells as a key source of this compensatory response. Both basal (K14+) and luminal (K8+) ductal cells contribute to regeneration through coordinated proliferation and migration, contributing structural reconstruction of the CVP epithelium prior to full stem cell recovery. Lineage tracing further demonstrates that these cells contribute to both the crypt region and newly formed taste buds, supporting their functional integration into the regenerating epithelium. These findings are consistent with previous studies demonstrating epithelial plasticity and regenerative contributions from non-canonical progenitor populations in glandular tissues 38.

Sox10+ ductal cells represent a distinct epithelial population with minimal overlap with Sox2+ and Sox9+ progenitors. Pseudotime analysis indicates that Sox10 expression is enriched at early stages of the regenerative trajectory, preceding Sox2 and Sox9 activation. Together, these findings suggest that Sox10+ cells function as an upstream, injury-responsive reserve population that transiently acquires progenitor-like properties following Lgr5+ stem cell loss. This interpretation is consistent with prior lineage tracing and single-cell studies demonstrating Sox10+ ductal cells as progenitors contributing to taste bud regeneration 12, 13.

Basement membrane restoration occurred rapidly following Lgr5+ cell ablation, before the full re-establishment of taste bud structures. This temporal separation suggests that basement membrane reconstruction is not a direct driver of taste bud regeneration, but rather reflects the re-establishment of a permissive niche environment required for subsequent regenerative processes. Although the specific cellular contributors to basement membrane repair cannot be definitively identified from the present data, the rapid recovery implies involvement of epithelial-adjacent populations, including duct-associated cells and surrounding stromal components. Importantly, the delayed onset of epithelial regeneration, together with the emergence of Sox10+ cells in duct-associated regions, indicates that regeneration is not solely a consequence of niche restoration, but involves active contributions from distinct progenitor populations. These findings support a model in which basement membrane restoration provides a supportive structural and signaling framework, while Sox10+ duct-associated cells function as a key cellular contributor to epithelial regeneration following Lgr5+ cell loss.

In addition, the transcriptomic analyses presented in this study are primarily descriptive and should be interpreted in the context of global transcriptional dynamics, which may also be influenced by changes in cellular composition during regeneration. Therefore, the observed transcriptional changes cannot be interpreted as cell-intrinsic regulatory mechanisms. Our data do not provide direct evidence for stable lineage reprogramming or permanent changes in cell identity. In particular, the scRNA-seq analyses are based on previously published datasets and are therefore interpreted as providing supportive, rather than direct, evidence for cellular relationships during regeneration. Instead, the observed changes are more consistent with transient, injury-induced cellular plasticity. This interpretation is supported by the dynamic and reversible nature of marker expression, as well as the temporal sequence of regeneration observed following Lgr5+ cell ablation.

Together with lineage tracing results, this suggests that VEG-derived cells are not a separate stem cell pool, but rather lineage-related epithelial cells that undergo transient reprogramming to restore the Lgr5+ niche. This mechanism aligns with injury-induced plasticity described across multiple epithelial systems, where committed or quiescent cell populations revert to stem-like states to support regeneration 1-4, 39. In this context, the CVP-VEG system represents a unique model of cross-compartment epithelial compensation, in which anatomically adjacent tissues dynamically contribute to regeneration following severe stem cell depletion.

Organoid formation assays further underscore the central role of Lgr5+ cells in initiating epithelial growth. When DT was applied at the time of organoid establishment, no organoids formed, indicating that Lgr5+ cells are indispensable for organoid initiation. In contrast, when DT was applied after 5 days of culture, a subset of organoids survived, coinciding with the emergence of Sox10+ cells within the organoids. These findings indicate a functional hierarchy in which Lgr5+ cells are required for organoid initiation, whereas Sox10+ cells contribute to epithelial maintenance under conditions of stem cell depletion. This interpretation is consistent with the transient contribution of K8-derived cells *in vivo*.

Collectively, our findings identify lineage plasticity and transient cellular reprogramming as a fundamental backup regenerative strategy that preserves epithelial homeostasis following severe stem cell loss. This plasticity-driven mechanism provides a conceptual framework for how epithelial integrity is maintained under acute injury and offers broader insight into regenerative processes across organ systems.

## Conclusion

Our study provides evidence for the existence of a compensatory epithelial cell population within VEG ducts that contributes to the regeneration of the CVP epithelium following loss of Lgr5+ stem cells. This injury-induced plasticity advances our understanding of epithelial regeneration and cellular dynamics in the tongue. Importantly, our findings highlight the regenerative potential of ductal epithelial cells and suggest that these cells may serve as therapeutic targets for taste disorders associated with epithelial injury.

## Supplementary Material

Supplementary figures and table.

## Data Availability

All data associated with this study are present in the paper or the Supplementary Materials.

## Abbreviations

CVP: circumvallate papilla
VEG: von Ebner's gland
DT: diphtheria toxin
DTR: diphtheria toxin receptor
Lgr5: leucine-rich repeat-containing G-protein coupled receptor 5
K8: keratin 8
K14: keratin 14
K13: keratin 13
HBCs: horizontal basal cells
GBCs: globose basal cells
PCNA: proliferating cell nuclear antigen
TEM: transmission electron microscopy
HE: hematoxylin and eosin
IF: immunofluorescence
RNA-seq: RNA sequencing
scRNA-seq: single-cell RNA sequencing
GO: Gene Ontology
TOR: target of rapamycin
Wnt: Wingless-related integration site
RT-qPCR: reverse transcription quantitative polymerase chain reaction
BrdU: 5-bromo-2-deoxyuridine
IdU: 5-iodo-2′-deoxyuridine
tdT: tdTomato
CreERT2: tamoxifen-inducible Cre recombinase
PBS: phosphate-buffered saline
MMP9: matrix metalloproteinase 9
ROCK1: Rho-associated coiled-coil containing protein kinase 1
Zo-1: zonula occludens-1
PGP9.5: protein gene product 9.5
Tuj1: class III β-tubulin
LEC: lingual epithelial cell
TBC: taste bud cell
UMAP: Uniform Manifold Approximation and Projection
