# Supplementary material for: Plasticity-Driven Regeneration of Circumvallate Papilla After Lgr5+ Stem Cells Loss
Source: Int J Biol Sci. 2026 May 11;22(10):5263–82. doi: 10.7150/ijbs.130382 (PMC13215247; doi:10.7150/ijbs.130382)
Supplement: Supplementary file 1 — Supplementary figures and table. [file ijbsv22p5263s1.pdf]

Supplementary Figures

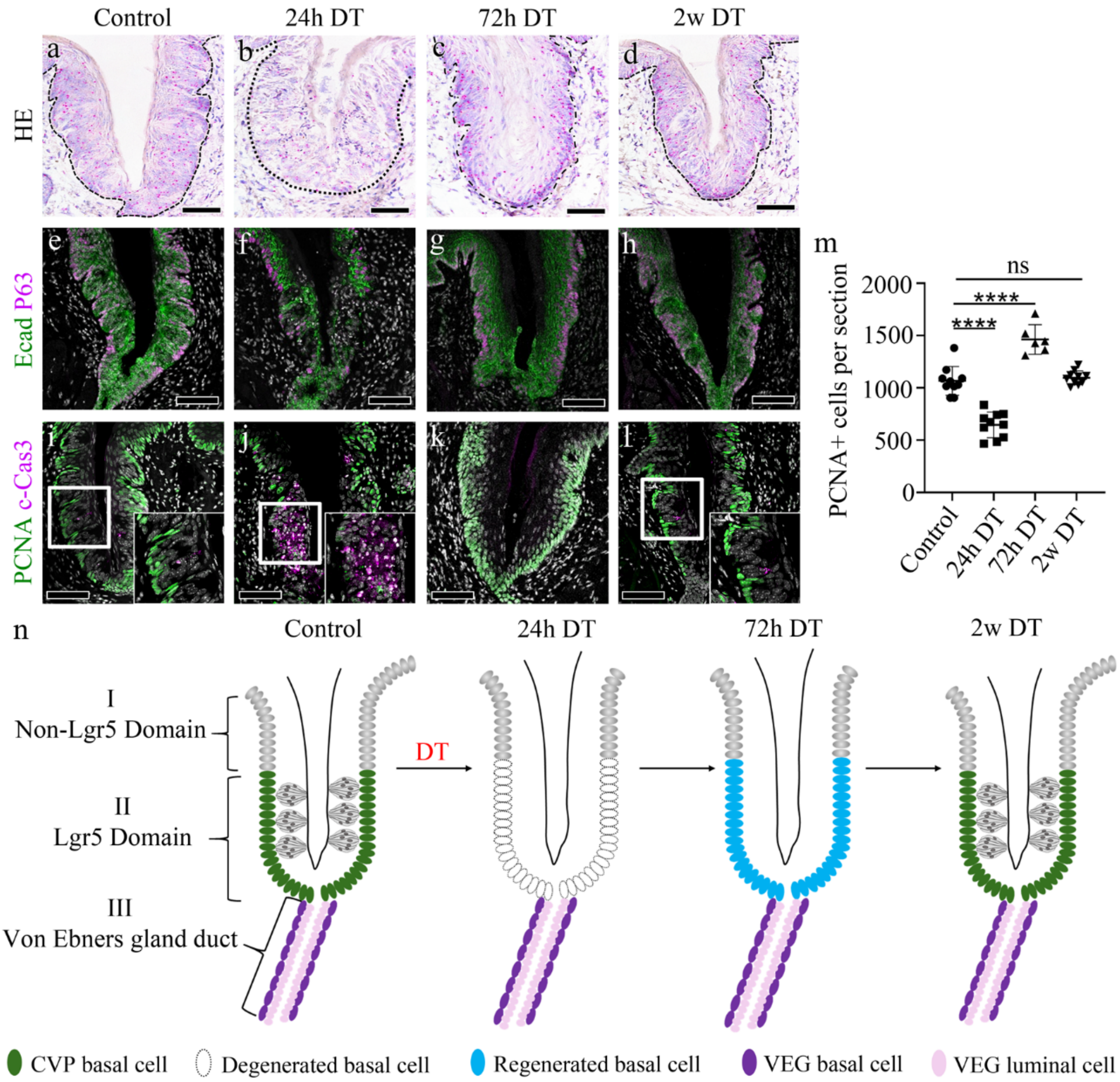

Supplementary Fig. 1: Ablation of Lgr5+ cells result in degeneration of basal cells and epithelial junction by apoptosis.

a, *Lgr5* mRNA is shown as red dots in the cells of the CVP trench in the control sample. **b**, 24 h after DT treatment, basal cells were disrupted, and very few cells expressing *Lgr5* mRNA were observed. **c**, At 72 h, basal cells regenerated, and Lgr5-expressing cells were observed in the basal layer. **d,** 2 w after DT treatment, Lgr5 was expressed in the CVP epithelium, similar to that in the control. **e**, Intact epithelial junctions with basal cells were observed in control mice, as indicated by E-cadherin and P63 staining, respectively. **f**, E-cadherin is weakly localized in the CVP epithelium, indicating loss of the epithelial junction. No p63 staining is observed, which shows degeneration of basal cells in the taste-bud-forming region. **g**, E-cadherin and P63 expression was observed in the CVP, indicating the regeneration of epithelial junctions and basal cells. **h**, E-cadherin+ and P63+ cells were observed in the CVP epithelium, as they were in the control. **i**, PCNA+ proliferating basal cells are observed in the CVP, along with a small number of cleaved caspase3+ apoptotic cells. **j**, The number of c-caspase3+ cells markedly increased in the taste-bud-forming region 24 h after DT treatment. **k,** PCNA+ proliferating cells were observed along the basal layer, with no apoptotic cells. **l**, Proliferating basal cells were observed around the taste buds with a few apoptotic taste receptor cells. **m**, The quantification results indicated that the number of proliferating PCNA+ cells was reduced 24 h after DT treatment, increased beyond control levels at 72 h, and then returned to control levels after 2 w. **n**, The schematic figure illustrates the degeneration of basal cells 24 h after DT treatment, followed by the regeneration of basal cells 72 h after DT treatment, and the regeneration of taste buds 2 w after DT treatment. Scale bar: a-d, 50 µm; e-l, 75µm

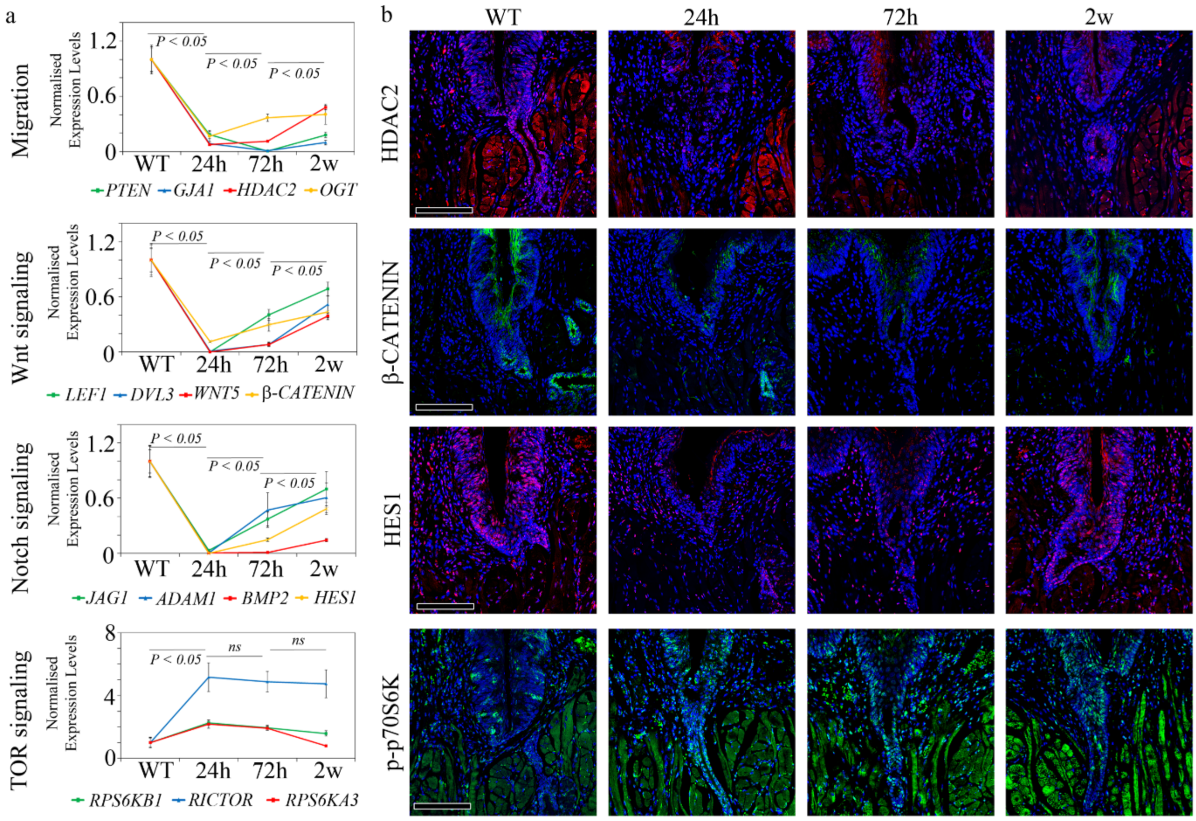
a
b
Migration
Wnt signaling
Notch signaling
TOR signaling
Normalised Expression Levels
P < 0.05
ns
WT
24h
72h
2w
PTEN
GJA1
HDAC2
OGT
LEF1
DVL3
WNT5
β-CATENIN
JAG1
ADAM1
BMP2
HES1
RPS6KB1
RICTOR
RPS6KA3
HDAC2
β-CATENIN
HES1
p-p70S6K

Supplementary Fig. 2: RT-qPCR and immunofluorescence validation of signaling changes during CVP regeneration after Lgr5+ cell ablation. (a) RT-qPCR analysis of representative genes associated with migration, Wnt signaling, Notch signaling, and TOR signaling in CVP tissue from control (WT) and DT-treated mice collected at the indicated time points. Migration-related genes (*PTEN*, *GJA1*, *HDAC2*, and *OGT*) were reduced after injury. Wnt-related genes (*LEF1*, *DVL3*, *WNT5A* and *β-CATENIN*) and Notch-related genes (*JAG1*, *ADAM10*, *BMP2* and *HES1*) were transiently downregulated at 24 h and showed recovery during regeneration. TOR-related genes (*RPS6KB1*, *RICTOR*, and *RPS6KA3*) showed early induction after DT treatment. (b) Immunofluorescence staining of HDAC2, β-catenin, HES1, and p-p70S6K in the CVP at the indicated time points. HDAC2, β-CATENIN, and HES1 signals were decreased after DT treatment and gradually restored during regeneration, whereas p-p70S6K signal was enhanced during the early regenerative phase. Data are presented as mean ± SD. Statistical significance was determined using one-way ANOVA followed by Tukey's multiple comparisons test. n = 6. Scale bars, 100 μm.

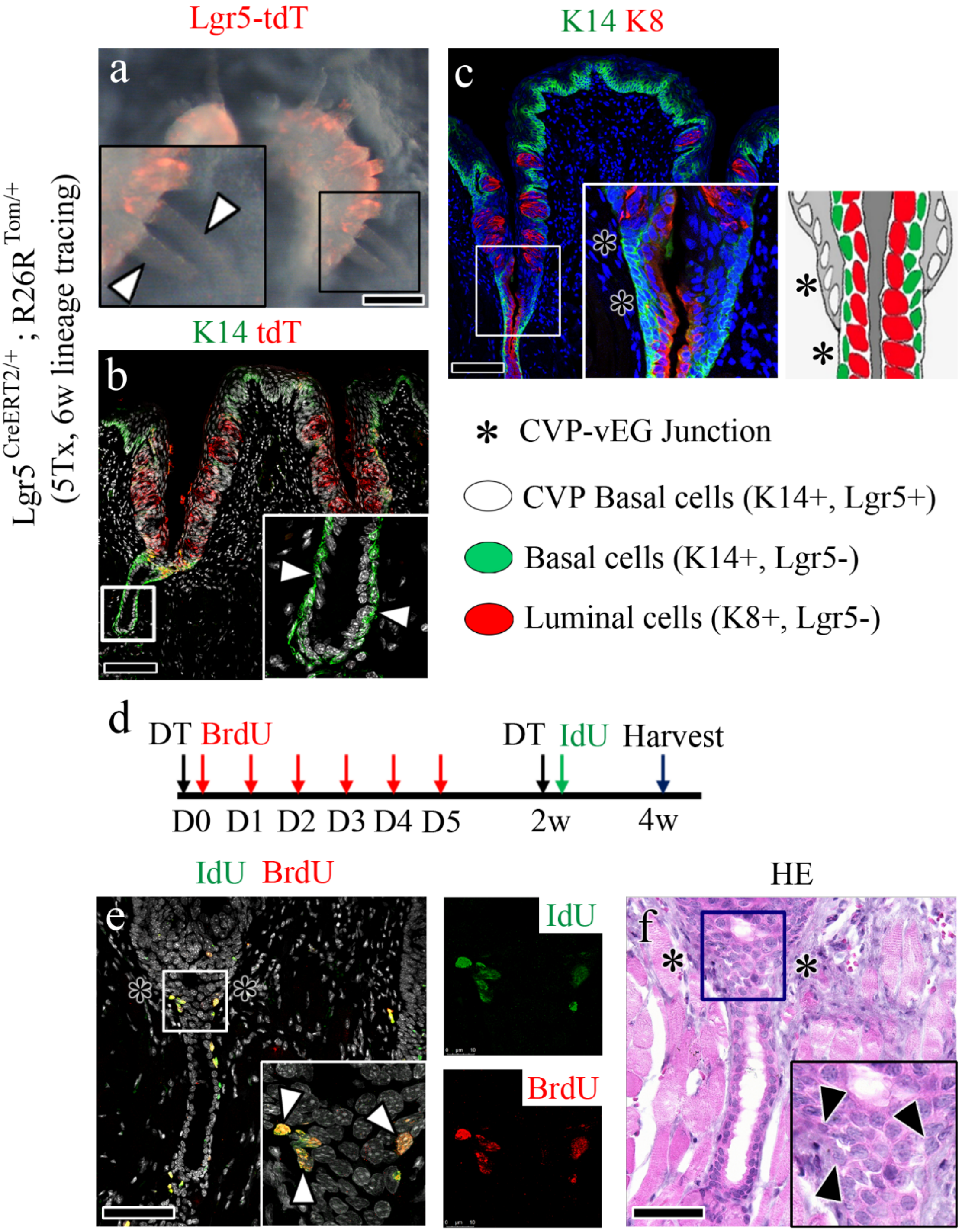
Lgr5 CreERT2/+ ; R26R Tom/+
(5Tx, 6w lineage tracing)
Lgr5-tdT
a
K14 tdT
b
K14 K8
c
* CVP-vEG Junction
CVP Basal cells (K14+, Lgr5+)
Basal cells (K14+, Lgr5-)
Luminal cells (K8+, Lgr5-)
d
DT BrdU
DT IdU Harvest
D0 D1 D2 D3 D4 D5
2w
4w
IdU BrdU
e
IdU
BrdU
HE
f

Supplementary Fig. 3: Lgr5 does not contribute to the homeostasis of ductal cells and ductal cells contain label-retaining cells

a, b, Whole-mount and sectioned views of $Lgr5^{CreERT2/+}$; $R26R^{Tom/+}$ mouse CVP after 6 w of lineage tracing. Lineage tracing of Lgr5+ cells for 6 w in $Lgr5^{cre}$; $R26^{tdT}$ mice indicated that Lgr5+ cells did not give rise to ductal cells during homeostasis. K14 and tdT staining showed that Lgr5+/ tdT+ cells were not observed in the VEG duct, but were widely detected along the CVP epithelium in the taste-bud-forming and crypt regions. Arrowheads indicate the absence of tdT+ cells in the duct of the VEG, both in the whole-mount and the section view. **c**, Immunofluorescence staining of the CVP with its associated VEG, showing K14-expressing basal cells and K8-expressing taste buds in the CVP papilla and K14+ basal cells and K8+ luminal cells in the VEG. Diagram showing K8+ and K14+ populations in the VEG duct. **d,** Experimental timelines of mice treated with DT, BrdU, or IdU. Mice were injected with DT, followed by five daily injections of BrdU to label the quiescent population of cells activated during injury. BrdU was injected with DT, and after 2 w, IdU was injected with DT and chased for 2 w. **e, f,** A small number of BrdU and IdU co-localizing cells were detected in the VEG basal cell layer 2 w after IdU injection, indicative of a quiescent cell population that was reversibly activated upon injury, as observed in the immunofluorescence and HE sections. Scale bar: a, 250 μm; b, c, 100 μm; e, f, 75 μm

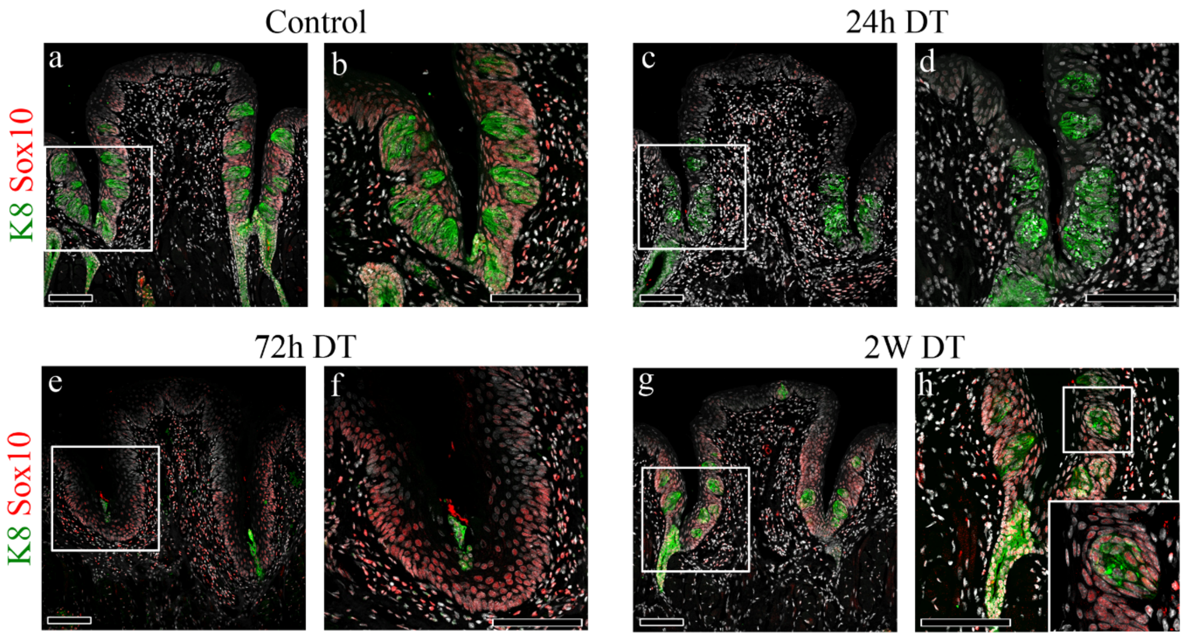

Supplementary Fig. 4. Spatial and temporal distribution of Sox10+ cells during CVP regeneration following Lgr5+ cell ablation. (a, b) Under control conditions, Sox10+ cells are detected within taste buds, intermingled with K8+ taste receptor cells. (c, d) At 24 h after DT treatment, Sox10+ cells are markedly reduced in both the CVP epithelium and within the taste bud as the epithelial structure degenerates, while Sox10+ cells remain detectable in the underlying mesenchymal region. (e, f) At 72 h post-ablation, Sox10+ cells are predominantly observed in the CVP crypt epithelium, particularly in regions adjacent to the VEG duct during the early regenerative phase. (g, h) At 2 weeks after DT treatment, Sox10+ cells are detected within regenerated taste buds together with K8+ cells. Scale bars, 100 µm.

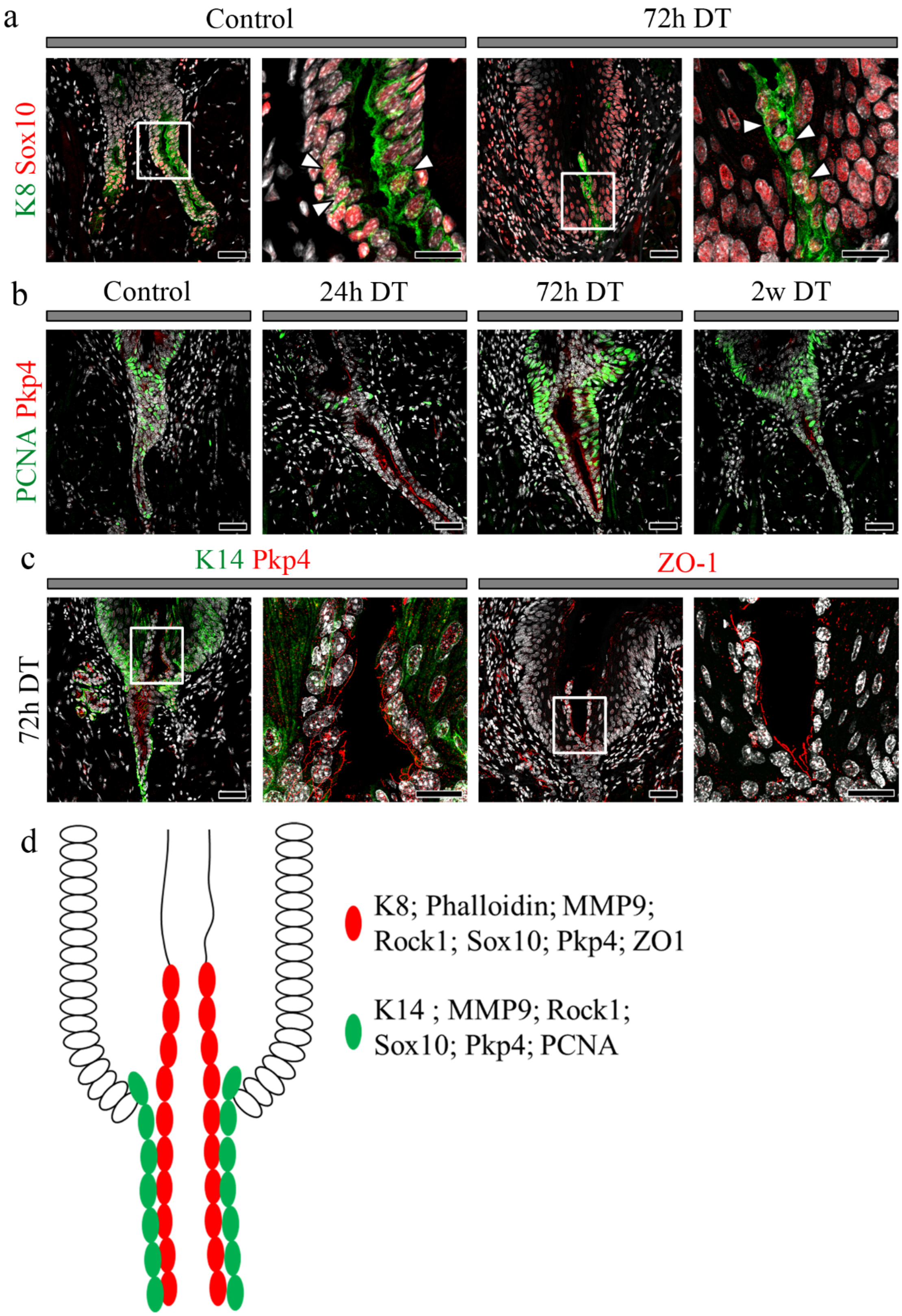
a
Control
72h DT
K8 Sox10
b
Control
24h DT
72h DT
2w DT
PCNA Pkp4
c
K14 Pkp4
ZO-1
72h DT
d
K8; Phalloidin; MMP9;
Rock1; Sox10; Pkp4; ZO1
K14 ; MMP9; Rock1;
Sox10; Pkp4; PCNA

Supplementary Fig. 5: Luminal cells are non-proliferative and express tight junction proteins

a, K8+ luminal cells expressed Sox10 in both control mice and in mice after 72 h of DT injection, indicating their ductal origin. **b**, Compared to other time points, 72 h of treatment resulted in more proliferative cells in the duct, marked by PCNA; however, the luminal cells arising in the groove were not proliferative, as they did not express PCNA. **c** Luminal cells in the groove express desmosomes and tight junction proteins, as represented by Pkp4 and ZO-1, respectively. **d** Schematic diagram of the different markers expressed by K8+ cells during migration. Scale bar: a,b,c 75 µm; a high magnification 50 µm; c high magnification, 25 µm.

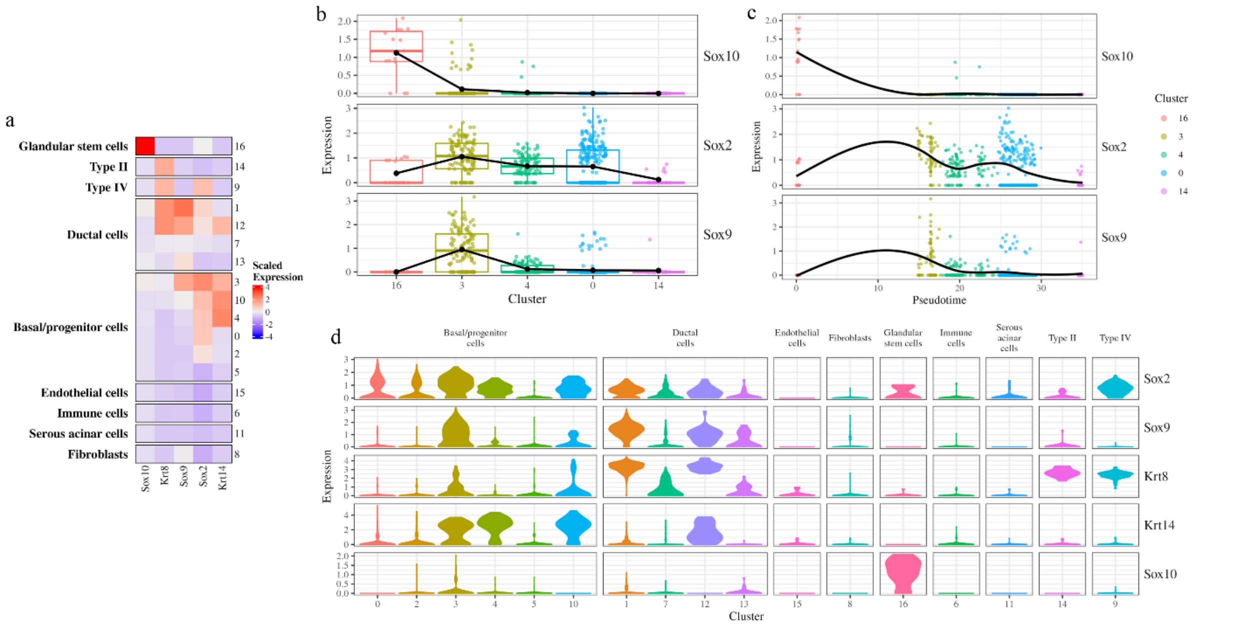

Supplementary Fig. 6. Single-cell RNA-seq analysis reveals a Sox10-enriched epithelial population with distinct transcriptional features in the CVP/VEG complex. (a) Heatmap showing scaled expression of representative marker genes across major cell populations. Sox10 expression is highly restricted to a specific cluster corresponding to glandular epithelial cells, whereas Sox2 and Sox9 are distributed across distinct cell populations. (b) Dot plot showing the expression levels and proportion of cells expressing Sox10, Sox2, and Sox9 across clusters. The Sox10-enriched cluster exhibits low expression of Sox2 and Sox9, indicating minimal overlap with canonical progenitor populations. (c) Pseudotime analysis showing dynamic expression patterns of Sox10, Sox2, and Sox9 along the inferred trajectory. Sox10 expression is enriched at early stages, while Sox2 and Sox9 expression increases at later stages. (d) Violin plots and feature plots showing the distribution of Sox2, Sox9, Krt8, Krt14, and Sox10 expression across clusters and annotated cell types. The Sox10-enriched population displays a distinct transcriptional profile with minimal overlap with Krt14-expressing basal progenitor cells and limited expression of Krt8-associated differentiated epithelial markers.

GOID	Description	Genes	Count	Adj. p-Value
GO:0050913	sensory perception of bitter taste	Rgs21, Tas2r106, Tas2r108, Tas2r109, Tas2r110, Tas2r114, Tas2r120, Tas2r129, Tas2r131, Tas2r136, Tas2r139, Tas2r140	12	8.99569E-09
GO:0050912	detection of chemical stimulus involved in sensory perception of taste	Tas2r106, Tas2r108, Tas2r109, Tas2r110, Tas2r114, Tas2r120, Tas2r129, Tas2r131, Tas2r136, Tas2r139, Tas2r140	11	8.61474E-08
GO:0050909	sensory perception of taste	Rgs21, Tas2r106, Tas2r108, Tas2r109, Tas2r110, Tas2r114, Tas2r120, Tas2r129, Tas2r131, Tas2r136, Tas2r139, Tas2r140	12	1.85136E-07
GO:0040013	negative regulation of locomotion	Arid4a, Bmpr1a, Braf, Ccl25, Cd200r1, Cygb, Dpp4, Epha4, Gja1, Gnrh1, Hdac2, Il33, Jag1, Krit1, Mia3, Muc2, Nr2f2, Ogt, Pdcd10, Prkg1, Pten, Rap2c, Rnf20, Scai, Sema3a, Stap1, Stk26, Wasl, Wnt5a	29	4.51357E-28
GO:2000146	negative regulation of cell motility	Arid4a, Bmpr1a, Braf, Ccl25, Cd200r1, Cygb, Dpp4, Epha4, Gja1, Gnrh1, Hdac2, Il33, Jag1, Krit1, Mia3, Muc2, Nr2f2, Ogt, Pdcd10, Prkg1, Pten, Rap2c, Rnf20, Scai, Sema3a, Stap1, Stk26, Wasl	28	1.20496E-27
GO:0030336	negative regulation of cell migration	Arid4a, Bmpr1a, Braf, Ccl25, Cd200r1, Cygb, Dpp4, Epha4, Gja1, Gnrh1, Hdac2, Il33, Jag1, Krit1, Mia3, Muc2, Nr2f2, Ogt, Pdcd10, Prkg1, Pten, Rap2c, Rnf20, Scai, Sema3a, Stap1, Stk26, Wasl	28	4.64102E-28
GO:0031929	TOR signaling	Bmt2, Cul3, F3, Foxp1, Gpr137c, Mapkapk5, Mfsd8, Mtm1, Nlk, Ogt, Pik3ca, Prkaa1, Pten, Rhebl1, Rictor, Rps6ka3, Rps6kb1, Sesn3, Syap1, Tbck, Ube2d1, Ube2n, Ube2w, Ube3a, Usp9x, Wac, Ywhaz	27	9.22117E-58
GO:0032006	regulation of TOR signaling	Bmt2, Cul3, F3, Foxp1, Gpr137c, Mapkapk5, Mtm1, Nlk, Ogt, Prkaa1, Pten, Rictor, Rps6kb1, Sesn3, Tbck, Ube2d1, Ube2n, Ube2w, Ube3a, Usp9x, Wac, Ywhaz	22	9.15966E-44
GO:0032008	positive regulation of TOR signaling	Bmt2, Cul3, F3, Foxp1, Gpr137c, Ogt, Rictor, Rps6kb1, Usp9x, Wac	10	1.54649E-17
GO:0016055	Wnt signaling pathway	Apc, Arl6, Bmp2, Brd7, Btrc, Cdc73, Csnk1g3, Ctnnd1, Cul3, Dvl3, Fzd3, Fzd5, Gnaq, Hdac2, Lef1, Lypd6, Map3k1, Myoc, Ndrg2, Nlk, Paf1, Pkd2, Prkaa1, Pten, Rnf138, Rtf1, Scel, Siah1a, Stk3, Tert, Tgfb1i1, Tlr2, Tmem170b, Ube2b, Wnt5a, Xiap, Zbtb33, Zranb1	38	3.73859E-66
GO:0030111	regulation of Wnt signaling pathway	Apc, Bmp2, Btrc, Cdc73, Csnk1g3, Ctnnd1, Gnaq, Hdac2, Lef1, Lypd6, Map3k1, Nlk, Scel, Stk3, Tert, Tlr2, Tmem170b, Ube2b, Wnt5a, Xiap, Zranb1	21	1.34941E-28
GO:0060070	canonical Wnt signaling pathway	Apc, Bmp2, Btrc, Csnk1g3, Ctnnd1, Dvl3, Fzd3, Fzd5, Gnaq, Hdac2, Lef1, Lypd6, Map3k1, Pten, Scel, Siah1a, Stk3, Tmem170b, Ube2b, Wnt5a, Xiap	21	6.46804E-29
GO:0007219	Notch signaling pathway	Adam10, Bmp2, Ccnc, Cd46, Fbxw7, Gas2, Ift74, Ift88, Jag1, Kit, Nkap, Pdcd10, Tspan15, Ttyh1	14	3.92934E-27
GO:0008593	regulation of Notch signaling pathway	Adam10, Ccnc, Cd46, Gas2, Jag1, Kit, Pdcd10, Tspan15	8	4.7076E-14

Supplementary Table S1. Gene sets contributing to GO terms in Figure 2.

List of genes associated with the GO biological processes presented in Figure 2. Each column corresponds to a representative GO term, and the genes included were identified by differential expression and enrichment analyses (adjusted $p < 0.05$).
